# Heavy metals contamination of seafood from the crude oil-impacted Niger Delta Region of Nigeria: A systematic review and meta-analysis

**DOI:** 10.1016/j.toxrep.2023.06.011

**Published:** 2023-06-20

**Authors:** Francis Uchenna Umeoguaju, Joyce Oronne Akaninwor, Eka Bassey Essien, Benjamin Achor Amadi, Chukwunonso Onyedika Igboekwe, Chimaobi James Ononamadu, Charles German Ikimi

**Affiliations:** a World Bank Africa Centre of Excellence in Public Health and Toxicological Research (PUTOR), University of Port Harcourt, PMB, Port Harcourt 5323, Rivers State, Nigeria; b Department of Biochemistry, Faculty of Science, University of Port Harcourt, Port Harcourt, Rivers State, Nigeria; c Department of Biochemistry and Forensic Science, Nigeria Police Academy, Maiduguri Road, P.M.B 3474, Wudil, Kano State, Nigeria; d Department of Biochemistry, Federal University Otuoke, Otuoke, Bayelsa State, Nigeria

**Keywords:** Heavy metals, Seafood, Fish, Niger Delta, Crude oil, Shellfish, Pb, Cd

## Abstract

This study aims at computing the pooled mean estimate (PME) and health risks of heavy metals in seafood obtained from the Niger Delta Region of Nigeria (NDRN), using data from existing literatures. Pubmed, Scopus and Google Scholar were searched to retrieve articles that investigated the heavy metal contents of edible seafood from the NDRN. Search hits were screened against predetermined criteria following which relevant data were extracted from eligible articles. The PME for each metal was computed by performing a maximum likelihood random effect model meta-analysis using the R Studio Software. Outcome from the meta-analysis involving 58 studies and a total of 2983 seafood samples revealed the following PMEs (mg/kg dry wt seafood) for the investigated heavy metals: As (0.777), Cd (0.985), Co (4.039), Cr (2.26), Cu (11.45), Fe (143.39), Hg (0.0058), Mn (13.56), Ni (5.26), Pb (4.35), and Zn (29.32). The health risk assessment suggests that seafood from this region poses considerable carcinogenic and non-carcinogenic risks to human consumers. Our finding calls for urgent actions aimed at identifying and eliminating point sources of heavy metals pollution of the NDRN marine environment. Inhabitants of NDRN are encouraged to reduce seafood consumption while diversifying their protein sources to include non-seafood options.

## Highlights

• Meta-analytic technique was used to compute PMEs of metals in seafood from NDRN.
• Seafood from the NDRN are rich sources of micronutrients like Cu, Cr, Mn, Zn and Fe.
• Seafood may pose a non-carcinogenic risk to consumers in NDRN due to Cd, Pb and Co.
• Seafood may pose a carcinogenic risk to NDRN consumers due to it’s As and Cr content.
• Reduction in seafood intake and diversification of protein sources is advised.

## Graphical Abstract

**Figure:**
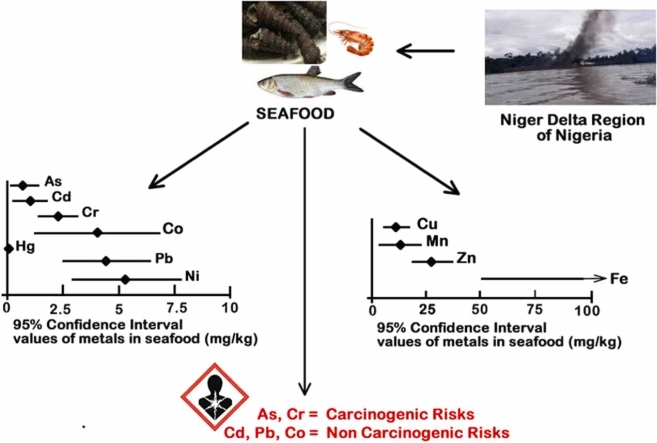
Heavy metal contents of seafood from the Niger Delta Region of Nigeria and its potential health risks.

## 1 Introduction

The Niger Delta Region of Nigeria (NDRN), which spans an area of about 110000 km^2^ and houses over 30 million inhabitants, is the hub of crude oil exploration activities in Nigeria [1]. The intensity of crude oil exploration activities in this region is evident from the fact that Nigeria derives over 80% of its national revenue from crude oil [1]. The NDRN is considered the largest delta in the world, consisting of several tributaries that empties into the Atlantic ocean through coastline that spans about 450 km [1], [2]. The region which comprises 9 states, has rich presence of several multinational oil prospecting companies, petrochemical industries as well as oil wells, flow stations and several kilometers of oil pipelines which crisscrosses the region [3], [4]. There is an extensive deterioration of the NDRN’s environment occasioned by several decades and annual records of oil spill events, intense oil bunkering and illegal artisan crude oil refining activities [1]. Over 13 million barrels of crude oil has spilled into this region between 1954 and 2012 with additional oils-spillage events occurring at about 200000 barrels per year [5], [6].

Seafood represent an important source of protein, vitamins (such as A, and E), minerals, essential fatty acids (such as omega 3 fatty acid) and livelihood to the inhabitants of this region [7], [8]. The nutritive potentials of seafood to the inhabitants of the NDRN is currently threatened by the degree of environmental pollution in this region [9]. The presence of pollutants such as heavy metals in aquatic habitats could adversely affect the population and diversity of marine species due to their tendencies to impair their reproduction and survival as well as encourage seafood migration [10], [11]. The potentials of the heavy metal pollutants to accumulate in seafood also expose human consumers of seafood to the toxic effects of these heavy metals [12]. Heavy metals are naturally occurring and persistent elements which find their way into several environmental matrices as a result of anthropogenic activities (such as mining, as well as industrial and domestic utilizations) or natural events such as volcanic eruptions. Some heavy metals such as Co, Cr, Cu, Fe, Mn and Zn perform essential cellular functions when ingested in trace quantities but triggers toxic effects when ingested in excess amount. On the other hand, exposure to heavy metals like As, Cd, Hg, Ni and Pb are known to exert only toxic cellular effects. For instance, exposure to Pb, Cd and As have been associated with increased risks of cardiovascular diseases in humans [13]. Pb and Cd also trigger neurological, cardiovascular, developmental, skeletal and reproductive toxicity upon exposure [14], [15]. Exposure to As, Ni, Co and Cr causes dermatological disorders while exposure to Hg have been associated with neurotoxicity, immunotoxicity and developmental toxicity [16].

The high anthropogenic activities in the NDRN, such as those due to crude oil exploration, non-oil industries and rapid urbanization, potentially favors the redistributions of heavy metal pollutants across different environmental matrices. For instance, during crude oil explorations, drilling machineries alongside with drilling fluids are used to drill down to deep underground crude oil reserves. Since heavy metals are constituents of crude oil, excavated earth and drilling fluids, [17], [18] crude oil exploration is therefore a potential source of heavy metal contamination of the NDRN. This line of reasoning is supported by observations that the environs of crude oil wells are contaminated with very high levels of heavy metals [20], [19]. Ohimain et al. [21] warned that if wastes emanating from oil exploration activities is not managed properly, they could be persistent contributors to the heavy metal burden of the NDRN. Overtime, following flooding, erosion, leaching or deliberate human activities, heavy metal from crude oil exploration sites, industrial effluents and municipal wastes finds their way into natural water bodies consequently increasing the heavy metal burden of these water bodies. Our previous study had reported high concentrations of heavy metals including Ni, Cd, Cr, and Pb in natural water bodies of the NDRN [4]. The increased presence of heavy metals in these water bodies suggests high possibility of the heavy metal contamination of seafoods from this region.

While individual studies have investigated metal levels in seafood from the NDRN [23], [24], [22], [25], [26], results from such studies have been inconsistent and so there is need for a comprehensive synthesis of these findings. A meta-analysis is needed to systematically analyze the existing data and determine the overall trends and variability in metal concentrations across multiple studies conducted in the region. To the best of our knowledge, there has not been any meta-analytic investigation of the heavy metal contents of seafood from NDRN. A meta-analysis of such nature would yield a more robust and precise pooled mean estimate (PME) which would afford a more reliable assessment of the health risks faced by inhabitants of this oil-rich region due to the presence of heavy metal in seafood. Furthermore, since factors such as seafood species, sampling locations, study period and anthropogenic activities around sampling sites are known to influence the elemental contents of seafoods [12], [27], [28], [29], [30], it is necessary to determine if these factors could explain some of the observed discrepancies in the heavy metal contents of seafood, as reported by different authors.

This study therefore aims at the systematic retrieval, the qualitative and meta-analytic synthesis of data extracted from studies that investigated the levels of any of the predominant heavy metals (i.e. As, Cd, Cr, Co, Cu, Fe, Mn, Hg, Ni, Pb, Zn) in seafood obtained from natural water bodies in the NDRN. The potential effect of seafood types, sampling locations, study year and anthropogenic activities around sampling sites on inter-study variations was also investigated. Outcome from this study would provide information on the potential health risks due to heavy metal exposure following seafood consumption as well as assist in the development of appropriate public health guidelines and regulations aimed at protecting inhabitants of the NDRN.

## 2 Methods

### 2.1 Database search and literature retrieval

This review and meta-analysis was carried out with adherence to the recommendation of the Preferred Reporting Items for Systematic Reviews and Meta-Analyses (PRISMA) guideline [31]. Three literature search engines (i.e. Pubmed, Scopus and Google Scholar) were extensively searched for articles that were published between 2000 − 2022. Articles of interest are those that reported on the heavy metal contents of seafood obtained from natural water bodies situated in the Niger Delta region of Nigeria. Separate search strategies were developed for each database using the database's specific search syntaxes. The search strategy combined keywords that describe the location of interest (such as "imo river", "imo state", "akwa ibom", "rivers state", "delta state", "Bayelsa state", "abia state", "ondo state", "edo state", "cross river", "calabar river", "niger delta") AND seafood types (including “seafood”, “fish”, “shellfish”, “oyster”, “periwinkle”, “shrimp”, “crab”, “catfish”, “tilapia”) AND heavy metals (such as "heavy metal", "trace element", "trace metal", copper, cadmium, zinc, nickel, chromium, arsenic, manganese, mercury, iron, cobalt, Pb). The exact strategy used to query Pubmed and Scopus are given below.

#### 2.1.1 SCOPUS search strategy

“( TITLE-ABS-KEY ( seafood OR fish OR shellfish OR oyster OR periwinkle OR shrimp OR crab OR catfish OR tilapia) AND TITLE-ABS-KEY ( "heavy metal" OR "trace element" OR "trace metal" OR copper OR cadmium OR zinc OR nickel OR chromium OR arsenic OR manganese OR mercury OR iron OR cobalt OR Pb OR Pb2 + OR Cr OR Cu OR Cd OR Zn OR Ni OR As OR Mn OR Hg OR Fe OR Co) AND TITLE-ABS-KEY ( "imo river" OR "imo state" OR "akwa ibom" OR "rivers state" OR "delta state" OR "Bayelsa state" OR "abia state" OR "ondo state" OR "edo state" OR "cross river" OR "calabar river" OR "niger delta" OR Nigeria))”.

#### 2.1.2 PUBMED search strategy

“(seafood OR fish OR shellfish OR oyster OR periwinkle OR shrimp OR crab OR catfish OR tilapia) AND ("heavy metal" OR "trace element" OR "trace metal" OR copper OR cadmium OR zinc OR nickel OR chromium OR arsenic OR manganese OR mercury OR iron OR cobalt OR Pb OR Pb2 + OR Cr OR Cu OR Cd OR Zn OR Ni OR As OR Mn OR Hg OR Fe OR Co) AND (Nigeria OR "imo river" OR "imo state" OR "akwa ibom" OR "rivers state" OR "delta state" OR "Bayelsa state" OR "abia state" OR "ondo state" OR "edo state" OR "cross river" OR "calabar river" OR "niger delta")”.

The search was initially conducted in October 2019 but was repeated in November 2022. The titles and abstracts of hits from the database searches were screened following which unique and relevant articles were selected for full text screening based on pre-defined inclusion criteria.

### 2.2 Eligibility criteria

Articles selected for this systematic review and meta-analysis were experimental in nature and determined heavy metals, namely As, Cd, Cr, Co, Cu, Fe, Mn, Hg, Ni, Pb, and Zn, in edible seafood sourced from natural water bodies within any of the 9 states (Rivers, Bayelsa, Edo, Delta, Cross River, Akwa Ibom, Ondo, Imo, Abia) of the NDRN. Therefore review articles, non-journal articles, non-English articles, articles that dwelled on farmed seafood, or seafood obtained from market far from water bodies or those from locations other than the states of NDRN were excluded from this study. Similarly, studies that were conducted on non-edible seafood, non-edible portions of seafood, those published before 2000, or those that reported wrong, ambiguous or unclear units, or whose full text was not available or retrievable online were excluded. Additionally, studies lacking key statistical details (i.e. sample size, SD or SEM) were excluded from meta-analysis. The selection of relevant article was carried out by FU and reviewed independently by CO and CI.

### 2.3 Assessment of study quality

The quality of the included studies were evaluated using a slight modification of the method of [4]. The procedure evaluated study quality across 5 domains comprising the quality of the testing equipment, the appropriateness of sampling and testing procedures, the completeness of results and statistical details, possible risk of sampling and detection biases and reportage of sampling location details, with each domain having multiple test criteria (Table 1). The test criteria were assigned a score weight of 1 or 2 based on the authors' assessment of the relative importance of each criterion in enhancing data quality. The test criteria in each domain of the checklist was completed as either “YES” or “NO” value, depending on the author’s judgment following each article’s assessment. Each article was screened against the entire quality checklist items during the evaluation process. All affirmative response were scored a value that corresponds to each criterion's score weight (i.e. either 1 or 2) while non-affirmative response were scored 0. To calculate percentage quality score for each study, we first summed the individual scores of each item of the checklist, and then expressed this total as a percentage of the maximum possible score.

**Table 1:** Study quality assessment checklist.

Quality Assessment Domains	Checklist Number	Test Criteria	Score weight
Testing Equipment	1	Was equipment validation reported?	2
	2	Was Validation/recovery data presented?	1
	3	Was instrument or method detection limit stated?	1
Sampling and testing procedures	4	#4 Was appropriate equipment used?	2
	5	^#5^ Was sampling done at appropriate sites?	1
	6	Were precautions for sample protection stated?	1
Results and statistical details	7	Was appropriate assay methodology used?	2
	8	Were sufficient statistical details provided?	2
	9	Were results presented with appropriate units?	2
	10	^#10^ Were sufficient details provided for the test outcome?	2
	11	Is the risk of attrition low?	1
Sampling and Detection Biases	12	Was blinding of sample collector stated?	1
	13	Was it stated that test samples were analyzed alongside with controls?	1
	14	Was blinding of assessor stated?	1
	15	Were random analyses of samples stated?	1
	16	^#16^ Was sufficient sampling performed?	2
Sampling location details	17	Were the anthropogenic activities at sampling sites stated?	1
	18	Were the GPS coordinates of sampling site stated?	1
	19	Was the sampling date (Year and month) stated?	1

Footnotes

^#4^ Under the "appropriate instrumentation" criterion, we checked if the instrumentation used for heavy metal determination was amongst those recommended by the Environmental Protection Agency for the respective metal (EPA, 1994).

^#5^ For this study, seafood obtained directly from its natural habitat or from fishermen was considered appropriate while those obtained from the market was inappropriate.

^#10^ Studies that did not indicate whether the result was presented in dry weight or wet weight basis were scored low in the “test outcome details" test criteria.

^#11^ Studies with discrepancies in the numbers of samples collected and the numbers analyzed is considered to have a high risk of attrition bias.

^#16^ Studies that collected up to three samples per sampling sites were considered to have collected sufficient samples.

### 2.4 Data extraction for qualitative synthesis

Information on the sampling and experimental methodology, study area description, heavy metals investigated, seafood investigated and results were carefully and methodologically extracted from each included study by one of the authors (FU) and then double checked by the other authors. This data was used to populate the summary table which had a predefined template that includes author’s last name and year of article publication, sampling locations and coordinates, anthropogenic activities, seafood type, sample size, heavy metals investigated and summary of outcome.

### 2.5 Data extraction for meta-analysis

Data from eligible studies including the mean, standard deviation (SD), sample size, seafood type (fish or shellfish), anthropogenic activities (i.e. oil industries, non-oil industries, urban or domestic), heavy metal type, year of study and seafood sampling locations (States and water bodies) were extracted from each study and entered into an excel spreadsheet template. Mean and/or SD data were computed for studies that reported only raw data or standard error of mean (SEM) values before insertion into the spreadsheet template. Data extraction from charts was accomplished using the pixel ruler software obtained from https://www.arulerforwindows.com. Heavy metals that were below detection limits were assigned the mean and SD values of 0.0001 and 0 respectively, to permit computation. Data inserted into the spreadsheet were those having the units of "mg/kg dry weight". Mean and SD of studies whose results were originally presented in “mg/kg wet” weight basis were converted to their dry weight equivalent by dividing the “mg/kg wet weight” values by a factor of 0.28. This factor corresponds to an average seafood moisture content value of 72% (N = 180 seafood samples, SD =8.12), as deduced from the data published by [32]. The authors of publications with missing data (such as missing SD, SEM, sample size, unclear or wrong units) were contacted by emails and those whose details were not clarified were excluded from the meta-analysis. Shortly before meta-analysis, mutiple entries from the same authors were combined together using the R’s implementation of the formula for combing mean and SD of similar groups [33] (i.e. (1a), (1b), (1c)).

(1a) $$N_{1}+\;N_{2}\;$$

(1b) $$\left(N_{1}M_{1}+N_{2}M_{2})/{(N}_{1}+\;N_{2}\right)$$

(1c) $$\surd (\frac{\left(N_{1}-1\right)SD_{1}^{2}+\left(N_{2}-1\right)SD_{2}^{2}+\frac{N_{1}N_{2}}{N_{1}+\;N_{2}}\left(M_{1}^{2}+M_{2}^{2}-2M_{1}M_{2}\right)}{N_{1}+N_{2}-1})$$

Eq. 1a combines sample size of two groups, Eq. 1b combines mean of two groups and Eq. 1c combines standard deviation of two groups. Where N_1_ and N_2_ are sample size of group 1 and group 2 respectively; M_1_ and M_2_ are mean of group 1 and group 2 respectively, SD1 and SD_2_ are the standard deviation of group 1 and group 2 respectively.

## 3 PME computation

The PME for each heavy metal reported in the included studies was computed by performing a meta-analysis on R Studio Software (version 3.6.1); [34]. In order to minimize the influence of inter-study variations on the PME, we assumed a maximum-likelihood random effect model for the meta-analysis [35]. The “meta” R studio package [36], was used for building the forest plots, funnel plot as well as performing the Egger’s test for publication bias while the “InfluenceAnalysis” function of the “dmetar” package [37] was used for performing the sensitivity analysis. The sensitivity analysis made it possible to identify and exclude highly influential studies from the meta-analysis. However, the results obtained prior to the exclusion of these highly influential studies were also presented.

### 3.1 Subgroup meta-analysis

We performed a subgroup meta-analysis to explore the potential sources of heterogeneity in the computed PMEs. Four subgroup categories including seafood type (i.e. fish or shellfish), anthropogenic activities at sampling sites (i.e. oil-industries or non-oil industries or urban or domestic activities), state-wise sampling locations (i.e. Rivers, Bayelsa, Edo, Delta, Cross River, Akwa Ibom, Ondo, Imo, Abia) and study year (i.e. 2005 and below, 2006 – 2010, 2011 – 2015, and 2016 – 2022) were examined. Information about the prevailing anthropogenic activities at sampling sites was obtained from the description given within each study. Any site with oil-related activities or with past history of oil spill was assigned “oil-related”, while non-oil related sites were assigned “non-oil related industries” or urban or domestic depending on the presence of non-oil industries, commercial or rural activities respectively.

### 3.2 Human health risk assessment

The potential non-carcinogenic and carcinogenic risks posed on the health of NDRN residents following the consumption of heavy metal contaminated seafood was investigated by calculating the target hazard quotient (THQ) and excess lifetime cancer risk [39], [38] using the PME derived from the meta analysis. To assess the non-carcinogenic risk, the THQ was computed by dividing the estimated daily intake (EDI) of each metal (through seafood consumption) by the oral reference dose (RfD) of each corresponding metal [40] (Equation 2).

THQ = 

(2) EDI x RfD

The EDI of each heavy metal (i.e. mg of metal per kg human body weight per day) was calculated by multiplying the heavy metal content of the seafood (i.e. mg/kg wet wt) by the food intake rate (FIR) (i.e. kg of food/person/day) and dividing by the average body weight of the individual (i.e. kg/person) as shown in Eq. 3 [38].

EDI (mg kg^-1^ day^−1^)=

(3) (FIRxHMseafood)/Bwt

Where FIR is the daily food intake rate (in kg/day), HMseafood is the concentration of heavy metal in seafood (in mg/kg wet wt) and Bwt is the Average body weight of adult human (kg).

The PME computed from meta-analysis was used as the metal content of the seafood. Since the PME values are in mg/kg dry wt, they were converted to mg/kg wet wt by multiplying by a factor of 0.28 (corresponding to 72% moisture, as previously described herein). A body weight of 60 kg was assumed as the average body weight of a normal Nigerian adult [41]. Using dataset from the World Bank’s Living Standards Measurement Study, Lo et al. [42] computed an average household seafood consumption rate of 0.974 kg/week (SD: 0.579) for inhabitants of southern Nigeria.This value is equivalent to a FIR of 0.0263 kg/day/person. An oral reference dose of 1.5, 3E-03, 1E-05, 0.14, 0.3 and 0.7 mg/kg/day were used for Cr (III), Cr(VI), Cd, Mn, Zn and Fe respectively while 3E-04, 1E-04, 3E-04 and 0.02 mg/kg/day were used for As, Hg, Co, Cu, and Ni respectively [43]. A value of 1.5E-04 mg/kg/day, derived from the interim reference level of Pb [44] was used for estimating risk due to Pb.

The carcinogenic risk was estimated using the Incremental Lifetime Cancer Risk (ILCR) (Equation 4). The ILCR estimates the probability of developing cancer following a daily exposure to a carcinogen for a lifetime of 70 years [45].

Incremental Lifetime Cancer Risk = .

(4) EDI x CSF

Where EDI (mg kg^-1^ day^−1^) is the lifetime estimated daily intake dose calculated as presented in equation 2, CSF (mg/kg-day)^-1^ is the oral cancer slope factor.

The oral cancer slope factors of 0.5, 0.0085 and 1.5 (mg/kg-day)^-1^ were applied for Cr (VI), Pb and As respectively [43].

## 4 Result

### 4.1 Literature search and study selection

**Fig. 1:**
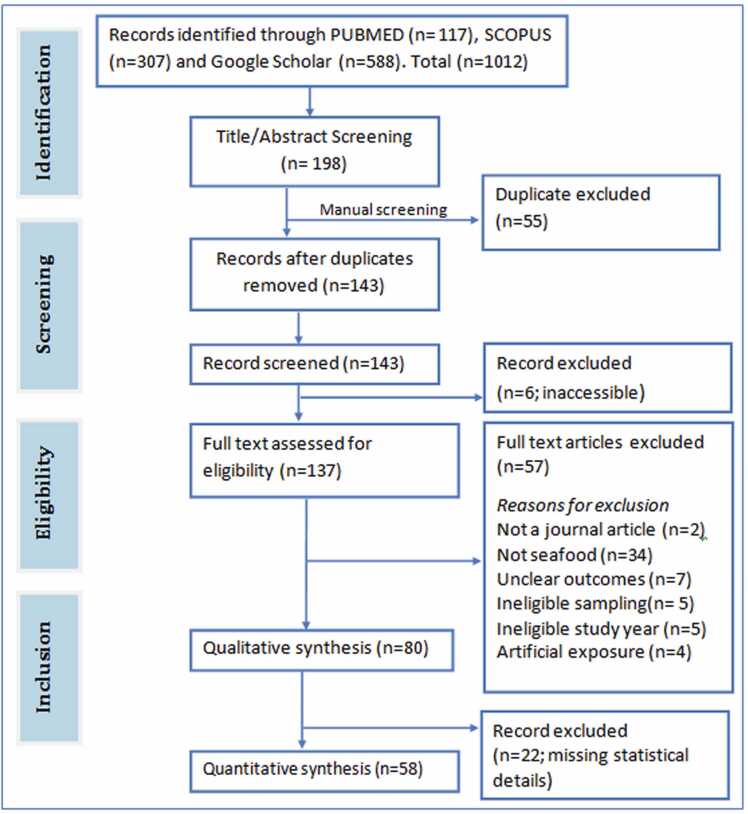
The PRISMA flowchart of the literature search and study selection process.

A total of 1012 hits were obtained following the searches conducted on Pubmed, Scopus and Google Scholar. Title and abstract screening yielded a total of 143 unique articles. Following the screening of these articles for eligibility, 63 articles did not meet the eligibility criteria and so were excluded while 80 and 58 of these articles were used for qualitative synthesis and meta-analyses respectively. (Fig. 1 PRISMA).

### 4.2 Study characteristics

The summaries of the included studies are presented in Table 2, Supplementary Table 1 S. These studies reported the levels of heavy metal including As, Cd, Co, Cr, Cu, Fe, Hg, Mn, Ni, Pb and Zn in edible portions of seafood obtained from natural water bodies that transverse the nine Niger Delta states comprising of Rivers, Delta, Bayelsa, Edo, Imo, Abia, Ondo, Akwa Ibom and Cross River state. Studies conducted in Rivers State obtained seafood from different communities along the stretch of New Calabar (such as the Iwofe, Aluu, Choba) and Bonny river (including the upper stretch, Okrika axis, Finima creek and Azuabie creek). Seafood samples were also obtained from water bodies within Ogoniland (such as the Kaa, B-Dere, Ogale and Bodo communities), Elele-Alimini, Bukuma, Oduoha, and Abuloma communities as well as from Andoni and Sombreiro River. In Delta state, seafood were sampled from the communities along the stretch of Warri River down to the coastal region as well as from Forcados, Ethiope, Orogodo, Aladja and Ofuafor Rivers. Seafood were also obtained from the Obotobe, Ubeji, Ekpan and Gbekobor Creeks. There are multiple presence of crude oil-related industries including oil prospecting firms, crude oil tanks, refineries, gas processing plants and petrochemical industries in many rural communities of Rivers and Delta States. Many of these industries generate wastes that end up in nearby water bodies. Several incidences of oil spill events, massive oil bunkering and illegal artisanal crude oil refining activities were reported in many of these communities. Water bodies such as the New Calabar River and Bonny Estuary, which flows through the densely populated Port Harcourt or Warri town respectively, were also reported to receive municipal and industrial wastes.

**Table 2:** Data abstraction table showing the summaries of included studies.

Author & year	Location / State	Analytic Instrument	N	Summary of outcome.	References
Ataikiru2008	Escravos River /Delta State	AAS	-	Cu (0.41–0.46), Cd (0.04–0.08), Cr (0.04–1.26), Pb (< 0.02–0.03), Ni (0.18–0.9) mg/kg dry wt	[46]
Omobepade2020	Ayetoro, Bijimi, Igbokoda and Obi axis of Ondo Coastal region /Ondo State.	AAS	-	Cd (0.002), Cu (0.013), Fe (0.0215), Pb (0.016), Ni (0.0013), Mn (0.0007), Zn (19.38) mg/kg dry wt	[47]
Ighariemu2018	Ikoli Creek /Bayelsa	AAS	-	Zn (23.74–32.61), Cr (BDL- 1.02), Pb (BDL - 0.83), Cd (BDL), Fe (66.36–85.54) mg/kg dry wt	[48]
Ibanga2021	Woji Creek /Rivers State	ICP-MS	2400	Mn (7–240), Fe (280–2400), Ni (1.4–4.2), Cu (4–80), Cd (0.05–0.07), Pb (0.3–2.5) mg/kg dry wt	[49]
Uwah2020	New Calabar River /Rivers State	AAS	4	As (BDL- 0.39), Cd (BDL- 0.26), Cr (5.66–10.73), Cu (12.33–20.28), Ni (5.66–9.03), Pb ( 6.92–8.11) mg/kg dry wt	[39]
Joseph2022	Qua Iboe River (along UAC, Iwuochang, Mkpanak Beach) /Akwa Ibom State	ICP-AES	180	Cr (0.011–0.44), Zn (8.4–23.4), Mn (0.10–11.5), Cd (0.021–4.1), Pb (BDL), Ni (0.010–20.2) mg/kg dry wt	[50]
Udo2012	Cross River Estuary /Cross River State	Flame photometer	18	Fe (270–1140), Zn (2.9–11.9), Cu (8.3–34.4), Mn (320–350), Pb (42.3–46.0) mg/kg dry wt	[51]
Oronsaye2010	Ikpoba River dam /Edo State	AAS	6	Fe (39.60–41.07), Cu (5.04–8.04), Mn (0.38–1.34), Zn (17.01–23.16), Ni (0.24–0.48), Cd (0.79–0.98) Cr(0.38–0.91), Pb (2.67–3.53) mg/kg dry wt	[52]
Opuene2008	Agbia/Nedugo to Polaku axis of Taylor Creek /Bayelsa State	AAS	5	Cd (0.08), Pb (0.350), Ni (0.156), Mn (3.736), Zn (2.516) mg/kg dry wt	[53]
Obasohan2008	Ogba River /Edo State	AAS	36	Cu (BDL- 7.79), Mn (BDL- 1.99), Zn (BDL- 8.9), Cd (BDL- 1.09), Ni (BDL- 1.01), Pb (BDL - 1.1) mg/kg dry wt	[54]
Obasohan2006	Ogba River /Edo State	AAS	72	Cu (BDL - 9.0), Mn (BDL- 1.14), Zn (BDL- 9.22), Cd (BDL- 0.29), Cr (BDL- 1.06), Ni (BDL - 1.02), Pb (BDL - 1.11) mg/kg dry wt.	[55]
Nwajei2002	Ofuafor River /Delta State.	AAS	20	Cu (0.15–0.33), Ni (1.65–2.44), Mn (1.46–1.83), Cr (1.65–2.60), Fe (15.48–21.85), Pb (0.32–1.12), Co (0.18–0.33) mg/kg dry wt.	[56]
Ayotunde2012	Stretch of Cross River (i.e. Ikom, Obubra, Calabar) /Cross River State.	AAS	120	Cu (0.001–0.031), Fe (0.043–0.71), Pb (0.003–0.005), Cr (BDL), Co (BDL), Cd (BDL- 0.004), Zn (0.003–0.006) mg/kg dry wt.	[57]
Inyang2002	Estuaries of Escravos, Forcados, Brass, Nun, Bonny, Andoni, Imo, Qua Iboe and Cross Rivers /Niger Delta Region	AAS	63	Cu (1.32–2.03), Pb (BDL- 1.33), Cd (BDL- 0.1), Fe (BDL-33), Zn (5.01–8.65), Ni (1.37–2.56), Cr (BDL), Mn (0.16–1.05), Co (BDL- 0.07), Hg (BDL- 0.05) mg/kg wet wt.	[24]
Eboh2006	Rivers in Oron Local Government Area /Akwa Ibom State	AAS	120–125	Cu (0.01–0.02), Zn (0.01–0.001), Pb (0.001–0.002), Hg (<0.001), As (< 0.001), Cr (<0.001), Cd (< 0.001) mg/kg wet wt.	[58]
Asuquo2004	Atabong west, Abana, Ine Oron, Esuk Idebe, Obufa Esuk, Henshaw Town Beach, Idundu, Ifeta, Mkpara Otop Ekpo, Mbiabo Okurikang regions of the Cross River System /Cross Rivers State	AAS	30	Cd (0.18–0.8), Pb (BDL- 10), Zn (11.3–63.1), Fe (11.3–63.1), Mn (BDL - 53), Cu (BDL- 3.0) mg/kg wet wt.	[59]
Asaolu2002	Ondo Coastal water /Ondo State	AAS	3	Pb (1.2–1.5), Ni (4.8 −5.8), Fe (13.8 −42.6), Cu (0.3–0.6), Zn (1.5–1.9), Cd (2.2 −21.3), Co (1.6 −3.8), Mn (22.5–41.3), Cr (2.7–6.1) mg/kg wet wt.	[60]
Asaolu2005	Igbokoda and Jirinwo axis of Ondo Coastal water /Ondo State	AAS	3	Pb (1.2–4.5), Ni (1.9–5.8), Cu (0.3–12.8), Zn (1.5–19.1), Cd (0.8–21.3), Co (1.6–3.8), Mn (22.5–41.3), Cr (2.7–6.1), Fe (13.8–78.4) mg/kg wet wt.	[61]
Wirnkor2021	Oguta Lake /Imo State	AAS	12	Fe (8.2–48.53), Cu (0.29 −0.61), Zn (3.86–7.10), Cr (BDL), Ni (0.12–0.48), Pb (0.27–0.70), As (0.17–0.733), Hg (5.93–13.13), Cd (0.06–0.489) mg/kg wet wt.	[62]
Patrick-Iwuanyanwu2022	Ka-Bangha River /Rivers State	AAS	20	Pb (3.35–11.54), Cd (0.17–1.53), Cr (3.12–10.357), Zn (8.15–38.41), Mn (7.14–34.85), Fe (88.97–308.18) mg/kg dry wt.	[63]
Aigberua2021	Okulu River /Rivers State	AAS	24	Fe (4–197), Zn (9.2 −35), Mn (0.2 −5), Cu (0–73), Cd (0–1.3), Pb (0 −54), Cr (0.00) mg/kg dry wt.	[64]
Ihunwo2022	Up and downstream regions of Woji Creek /River State	AAS	60	Cd (2.72–5.8), Pb (0.001–51.00) mg/kg wet wt.	[65]
Ihunwo2020	Woji Creek (Samberiro River), /Rivers State	AAS	80	Cd (3.86–24.62), Cr (BDL), Pb (2.60–10.11), Cu (3.13–33.48), Ni (BDL), As (BDL) mg/kg wet wt.	[23]
Oyibo2018	Ogulahan River Delta state, Forcados terminal of Ogulaha River /Delta State	AAS	21	Pb (BDL- 5.54), Hg (BDL), Cr (BDL- 2.69), Cd (BDL- 0.68), Fe (3.56–46.59), Zn (1.49–10.56), Cu (0.44–5.95), As (BDL- 0.54) mg/kg dry wt.	[66]
Agunbiade2011	Awoye axis of Ondo Coastal water /Ondo State.	AAS	20	As (0.09–0.1), Cd (0.28 – 0.29), Cr (4.38–4.92), Cu (18.72–24.49), Ni (0.56–0.58), Pb (0.29–0.33), Fe (199–212), Mn (284–293), Zn (96.7–106) mg/kg dry wt.	[22]
Okoro2007	Forcados River (Oguogbene, Aboro, Yokri) /Delta State	AAS	-	Ni (0.515–0.650), Pb (0.025–0.350), Cu (0.825–0.875), Hg (0.000–0.001), Cd (0.005–0.007) mg/kg wet wt.	[67]
Ossai2014	Warri River (Otokutu) /Delta State	AAS	-	Zn (20–34), Pb (0.45–0.97), Cu (0.82–1.39), As (0.4–0.83), Fe (0.42–0.8), Cd (0.39–0.76), Hg (0.3–0.64) mg/kg dry wt.	[68]
Olowoyo2010	Warri Coastal Water /Delta State	AAS	-	Pb (0.002–8), Ni (0.01–3.5), Fe (0.002–1.61), Cd (0.009–18.13) mg/kg dry wt.	[69]
Ogunola2017	Okrika /Rivers State	ICP-AES	48	Cr (3.4–4.06), Cu (1.11–16.32), Fe (148 −225.86), Ni (0.47–1.57), Zn (9.24–50.41) mg/kg dry wt.	[70]
Nkpaa2016	Kaa, B-Dere, Bodo in Gokana and Khana Local Government Area, Ogoniland /Rivers State	AAS	120	Cr ( 2.9–9.98), Cd (0.52–1.8), Pb (7.01–31), Zn (23–46), Mn (8.67–60.9), Fe (293–1040) mg/kg wet wt.	[38]
Nkpaa2017	Bodo City and B-Dere, Ogoniland /Rivers State	AAS	20	Cr (1.84–4.49), Cd (0.96–3.82), Pb (1.64–3.67), As (2.13–4.89), Ni (4.35–8.34 Fe (1256–2593 mg/kg dry wt.	[71]
Inam2012	Qua Iboe River Estuary /Akwa Ibom State	AAS	10	Fe (36.06), Ni (0.58), Pb (0.05), Cr (0.66), Cu (6.7), Zn (3.93) mg/kg dry wt.	[72]
Godwin2011	Azuzuama, Lobia, Ogboinbiri, Yenagoa /Bayelsa State	AAS	20	Cd (0.09 – 5.50), Co (5.10 – 11.70), Pb (1.20 – 61.20), Ni (2.10 – 13.0) mg/kg dry wt.	[73]
Davies2006	Upper limit of the Bonny Estuary /Rivers State	AAS	> 200	Cr (0.01–0.034), Cd (0.0001–0.002), Pb (0.009–0.015) mg/kg dry wt.	[74]
Abarshi2017	Bonny River and Finima Creek /Rivers State	AAS	120	Cu (0.001–0.031), Fe (0.043–0.071), Pb (0.003–0.005), Cr (BDL), Co (BDL), Cd (0–0.004), Zn (0.002–0.006) mg/kg dry wt.	[7]
Marcus2013b	Bonny River and Creeks around Okrika /Rivers State	AAS	18	Hg (0.002–0.09) mg/kg dry wt.	[75]
Ideriah2006	Upper Bonny River /Rivers State	AAS	-	Pb (4.39–9.45) mg/kg dry wt.	[76]
Freeman2017	Ekpan Creek, Warri /Delta State	ICP-MS, AAS	30	Zn (0.001–2.072), Fe (0.001–21.2), Pb (0.001–3.28), Cr (0.001–0.897), Cd (0.001–0.301), Mn (0.001–3.074), Cu (0.001–1.917) mg/kg wet wt.	[77]
Ezemonye2019	Benin River (Koko community) /Delta State	AAS	54	Mn (0 – 67), Fe (0–223.1), Cu (0–146), Cd (3.191), Ni (0 – 106), Pb (0–88), Co (0 −110.6), Zn (8.5–154.8) mg/kg dry wt.	[78]
Ezemonye2016	Benin River /Edo State	AAS	-	Mn (25 – 40), Fe (6 – 120), Cu (7 – 70), Cd (0.2), Ni (39 – 60), Pb (22 – 39), Co (20 – 35) Zn (50–85 mg/kg dry wt.	[79]
Etesin2007	Imo River /Imo State	ICP-MS	-	Zn (7.5 – 14), Cu (1–2.0), Pb (0.05 – 1), Cd (0.01–0.09) mg/kg dry wt.	[80]
Chindah2009	Bonny and New Calabar River /Rivers State	ICP-AES	-	Cr (0.002–0.130), Cd (0.0005–0.014), Pb ( 0.009–0.075), Zn (0.294 – 3), Cu (0.63–8.78) mg/kg wet wt.	[81]
Adebayo-Tayo2010	Itu and Oron Creeks (Oron, Itu LGA) /Akwa Ibom State	AAS	120	Fe (17.2 – 22.7), Zn (24.4 – 94.2), Pb (0.28 – 0.84), Cd (0.02 – 0.15), Mn (24.2 – 88.6), Cu (3.4 – 7.4) mg/kg wet wt.	[82]
Woke2016	Andoni River (Khana LGA) /Rivers State	AAS	30	Cd (0.01–0.04), Cu (25 – 30), Pb (0.2–0.55), Zn (22 – 42) mg/kg dry wt.	[83]
Ubiogoro2017	Egbokodo River in Warri, River Ethiope in Sapele, Urie River in Igbide Isoko, Asaba-Ase Creek, Aragba River in Abraka, and Uzere Creek /Delta State	AAS	6	Fe (11.3–24.03), Cu (0–0.09), Ni (0.07–0.89), Cd (0.12–0.26), Zn (3.74–7.83), Mn (0–0.57) mg/kg wet wt.	[84]
Owhonda2016	Bodo River, Ogoniland /Rivers State	XRF	-	Cr (10.3–10.5), Ni (8.7–10.2), Co (3.0 – 4), Zn (71.2–74.5) mg/kg dry wt.	[26]
Otitoju2013	Oron River, Itu River /Akwa Ibom State; Abuloma River /Rivers State	AAS	-	Cd (0.0423–0.107), Pb (0–0.276), Cu (0.3423–0.535), Cr (0), As (0), Hg (0) mg/kg dry wt.	[85]
Olowoyo2011	Warri Coastal water /Delta State	AAS	40	Pb (0.68–10.34), Ni (0.72 – 482), Fe (0.001–457.11), Cu (0.002 – 459), Zn (0.83–5.06), Cd (0.002–1.45), Mn (1.25–72.27), Cr (205.09 −1453) mg/kg dry wt.	[86]
Nwoko2015	Choba and Aluu Axis of the New Calabar River /Rivers State	AAS		Pb (0.097–0.655), Cu ( 17.7–58.2), Zn (28.5 −52.1), Cd (0) mg/kg dry wt.	[87]
Favour2014	Iwofe community of the New Calabar River /Rivers State	AAS	12	Fe (0 – 54), Pb (0 – 22), Cd (0 – 5) mg/kg dry wt.	[88]
Wokoma2014	Lower Sombreiro River /Rivers State	AAS	30	Fe (48.31–67.43), Cr (6.30–9.91), Cu: (2.7–5.31), Ni (2.89–4.17), Cd (0), Co (0.74–1.71), Pb (0.17–0.23), Zn (11.61–21.37) mg/kg dry wt.	[89]
Wegwu2006	New Calabar River /Rivers State	AAS	20	Hg (0), Pb (0.54–1.74), Cd (0.1–0.16), Cr (0.08–4.25), Cu (1.14–6.02), Zn (2.04–9.02), Fe (8.14–9.02) mg/kg wet wt.	[90]
Wangboje2017	Orogodo River (Mbiri, Agbor and Obazagbon communities) /Delta State	AAS	108	Pb (0.29–2.76), Cd (0.03–0.47), Cu (1.34 −10.57), Zn (14.08–91.62), Fe (3.5–286.1) mg/kg dry wt.	[91]
Wangboje2015	River Niger (Agenebode community) /Edo State	AAS	34	Pb (0–0.05), Cu (0.855–1.367), Cd (0–0.092), Zn (52.23–128.87) mg/kg dry wt.	[92]
Uhegbu2012	Ethiope River /Delta State	AAS	120	As (0.079–0.152), Cr (0.046–0.081) mg/kg dry wt.	[93]
Olusola2015	Ayetoro and Awoye communities of Ilaje LGA along coastal area of Ondo /Ondo State	AAS	15	Cr (0), Cd (0.31–0.34), Pb (0.01–0.08), Cu (0), Zn (0.31–0.51), Ni (0–0.02) mg/kg dry wt.	[94]
Ololade2011	Ondo Coastal water /Ondo State	AAS	180	Cd (0.57–29.39), Pb (0.02–47.61), Ni (0.1 −6.9) mg/kg dry wt.	[32]
Okogwu2019	Cross River System (Ndibe beach, Itigidi, Orah, Unwanna, Ehoma lake, Iyieke lake) /Cross River State	AAS	40	Fe (88.92 – 420), Zn (6.02–21.95), Cu (0.5 −2.6), Mn (0.05–2.81), Pb (0.1–0.3), Cr (0.2–0.5) mg/kg dry wt.	[25]
Oguzie2003	Ikpoba River Benin /Edo State	AAS	81	Cd (0.01–0.08), Cu (0.02–2.15), Pb (0.2–1.25), Zn (2.3–8.15) mg/kg dry wt.	[95]
Nwabueze2010	Obotobe and Gbekobor Creeks in Burutu Local Government Area /Delta State	AAS	24	Hg (0.153–1.3), Pb (1.38–5.383), Ni (0.22 −34.31), Cr (0.86 −3.775), Cu (3.250–10.32), Mn (0.563–4.149), Fe (16.9–48.94), As (1.7–3.51), Cd (0.911–1.349) mg/kg dry wt.	[96]
Nduka2006	Oduoha River (Emuoha LGA) /Rivers State	AAS	4	Pb (0.5 – 1), Zn (12.1–42.9), Cu (2.9–13.7), Ni (0.8–5.8), Cd (0.1–0.3), Co (0–0.9), Cr (0 −0.8), Fe (14.2–34.2), Mn (1–4.6) mg/kg wet wt.	[97]
Moslem2018	Azuabie Creek (Upper Bonny Estuary) /Rivers State.	AAS	120	Cr (0–7.5), Ni (0–4.59), Cu (1.36–56.35), Pb (0–7.07), Cd (0–1.01) mg/kg dry wt.	[40]
Moslen2017	Azuabie Estuarine Creek /Rivers State	AAS	96	Cr (0–8.25), Ni (0–5.68), Cu (0.93 – 34.95), Pb (0.88 −12.12), Cd (0–1.45) mg/kg dry wt.	[98]
Marcus2013a	Bonny River and Creeks near Okrika /Rivers State	AAS	240	Pb (0.028–4.675), Ni (0.038–81.275), Cd (0–1.375) mg/kg dry wt.	[99]
Howard2006	Mangrove swamp of Bukuma oil field /Rivers State	AAS	150	Zn (1.3–4.53), Pb (0.14–0.3), Cd (0.01–0.06), Cu (0.07–0.25), Ni (0.07–0.12) mg/kg dry wt.	[100]
Ekpo2008	Ikpoba River /Edo State	AAS	30	Pb (0–0.004), Cd (0.001–0.002) mg/kg wet wt.	[101]
Daka2008	Azuabie creek (Upper Bonny Estuary) /Rivers State	AAS	14	Cd (0.01–0.06), Pb (0.104–0.31) mg/kg dry wt.	[102]
Ajima2015	Mbaa River /Imo State	AAS	36	Pb (3.57–5.40), Zn (2.21–2.24), Cd (0.3–0.59), Cu (2.61–3.65), Fe (14.68–17.19) mg/kg wet wt.	[103]
Abarikwu2017	Ogale, Elele Alimini /River State	AAS	20	Ni (0.4–0.6), Cd (0.2–0.32), Zn (30–60), Cr (2–4), Pb (3) mg/kg dry wt.	[104]
Akankali2018	New Calabar River /Rivers State	AAS	-	Pb (7–31.96), Cd (< 0.001), Zn (126–158), Hg (< 0.001) mg/kg dry wt.	[105]
Omuku2008	Owah-Abbi (Ethiope) River /Delta State	AAS	-	Pb (0.14–0.34), Cd (0.05–0.19), Cr (0.05 −0.13), Zn (0.18–0.31), Cu (0.15–0.41) ppm wet wt.	[106]
Edem2009	Henshaw Townbeach market Calabar South /Cross River State	AAS	20	Pb (0.062), Zn (0.095), Cd (0.017), As (0), Hg (0) ppm (dry wt.)	[107]
Edet2014	Itu River /Akwa Ibom State	AAS	24	Cu (0.005–0.063), Fe (0.05–0.13), Zn (0.007–0.123), Cd (0–0.011), Hg (0), Pb (0) ppm dry wt.	[108]
Alinnor 2005	Aba River /Abia State	AAS	15	Zn (4.17 – 8), Ni (0–0.88), As (0–0.008), Hg (0–0.026), Co (0–0.06), Mn (0–0.865) ppm dry wt.	[109]
Ediagbonya2019	Oluwa River (Okitipupa) /Ondo State.	EDXRF	12	Cr (3.1–6.4), Mn (166.3 −244.4), Fe (1200–1700), Co (6.50–13.1), Ni (2.4–5.3), Cu (396.8 −492), Zn (298.9–492.3), As (3.1–7.4) mg/kg dry wt.	[110]
Enuneku2017	Koko Town axis of Benin River /Edo State	AAS	18	Zn (0.29–1.10), Cd (0.07–0.32), Pb (0.02–0.1), Mn (0.06–0.25), As (0.05–0.25), Ni (0.09–0.4) mg/kg dry wt.	[111]
Enuneku2015	Ubeji Creek,Warri /Delta State	AAS	9	Fe (55.50–158.90), Mn (2.55–7.23), Zn (15.10–37.20), Cu (1.11–2.42), Cr (0.12–0.77), Cd (0.61–0.95), Ni (0.35–1.15), Pb (0.57–1.48) mg/kg dry wt.	[112]
Nduka2010	Aladja River; Warri /Delta State.	-	5	Mn (0.15–3), Cd (<0.01, 0.2), Cr (0.01, 0.8), Zn (1.04–10.8), Ni (0.01, 6.82), Fe (0.8–18.01), Pb (0.01–2.4) mg/kg wet wt.	[113]
Obasohan2007a	Ogba River /Edo State	AAS	2	Cu (14.53–18.04), Mn (0.94 −3.50), Zn (14.93 −15.16), Cd (0.10 −0.11), Cr (1.02–3.96), Ni (0.28–0.86), Pb (2.67–4.00) mg/kg dry wt	[114]
Obasohan2007b	Ikpoba River /Edo State	AAS	36	Cu (4.101–4.4), Mn (0.5–0.88), Zn (5.8–6.22), Cr (0.734–0.883), Ni (0.071–0.174), Pb (1.972–2.223) mg/kg dry wt.	[115]

Abbreviations

AAS: Atomic Absorption Spectroscopy, BDL: Below Detection Limits, EDXRF: Energy Dispersive X-ray Fluorescence, ICP-AES: Inductively coupled plasma-atomic emission spectrometry, ICP-MS Inductively Coupled Plasma Mass Spectrometry, N: Sample size, XRF: X-ray Fluorescence

The studies conducted in Bayelsa obtained their seafood from Azuzuama, Lobia and Ogboinbiri communities in Yenagoa. The presence of oil exploration industries and urban centers have been reported around these locations. In Edo state, seafood samples were largely collected from Ikpoba and Benin Rivers as well as from Agenebode community (along the River Niger). These water bodies are potentially contaminated with wastes from both crude oil and non-crude oil related industries. Studies conducted in Akwa Ibom obtained seafood samples from Itu, Oron and Qua Iboe River while those conducted in Imo obtained seafood from Mbaa and Imo Rivers. These water bodies especially those of Imo, Mba and Qua Iboe Rivers receives effluent from crude and non-crude industries, agricultural runoffs and municipal wastes. Aba River is the sampling location of included studies conducted in Abia state while those conducted in Cross river state, were sampled from the Cross-river system (consisting of the Ndibe beach, Itigidi, Orah, Unwanna, Ehoma lake and Iyieke lake). Activities around sampling sites of the Aba river and Cross-river system are mainly those of non-crude oil related industries. Seafood used for studies conducted in Ondo state were obtained from communities along the Ondo coastal area including Ayetoro and Awoye as well as from Oluwa River. Farming and fishing were the predominant activities around most of the sampling site while some of the sites had oil exploration activities. The seafood investigated in the included studies consists of different species of commonly consumed fish (including Tilapia, Catfish, Bonga shad, Carp, Croaker, Mackerel, Sardine, mudskipper, mullets and others) and shellfish (Clam, Crayfish, Shrimp, Oysters and Periwinkle) (Supplementary Table 1 S).

### 4.3 Study quality

**Fig. 2:**
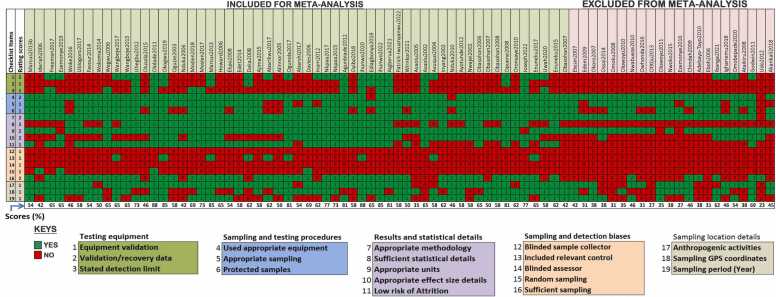
Performance of included studies across each assessment test criteria.

**Fig. 3:**
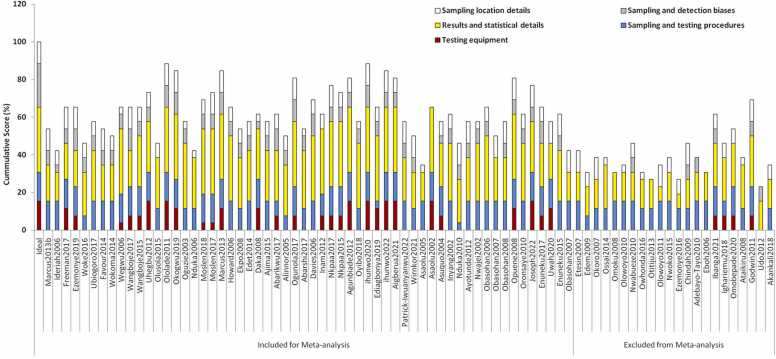
Cumulative performance of included studies across each assessment domains and across all assessment domains.

The majority of the included studies performed poorly in the "Testing equipment" and "Sampling and detection bias" domains of the quality assessment (Fig. 2). Most of the studies did not report any data to suggest that instrument or method validation was performed before analysis. Instruments or method detection limits of investigated heavy metals were also not reported in most of the included studies. Mention was not made in almost all the included studies, on efforts made to minimize detection and sampling bias. The sampling and testing procedures reported in most of the studies were okay while only about two-third of the studies presented sufficient result details and statistical details. Overall, the studies used for meta-analysis had an average percentage cumulative score of 62.7 while those excluded from meta-analysis (due to insufficient statistical details) had an average cumulative score of 39.2 (Fig. 3).

### 4.4 Pooled mean estimates (PME) of heavy metals in seafood

**Fig. 4:**
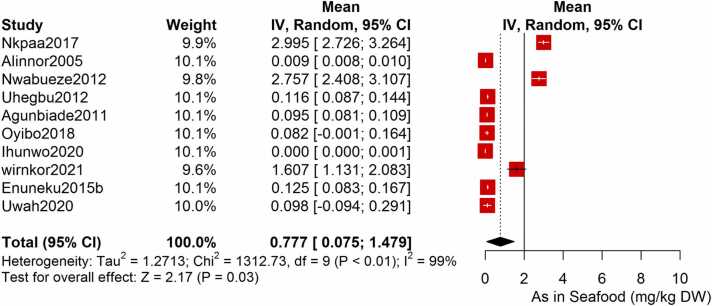
Forest plot representation of the meta-analysis of the As contents of seafood.

**Fig. 5:**
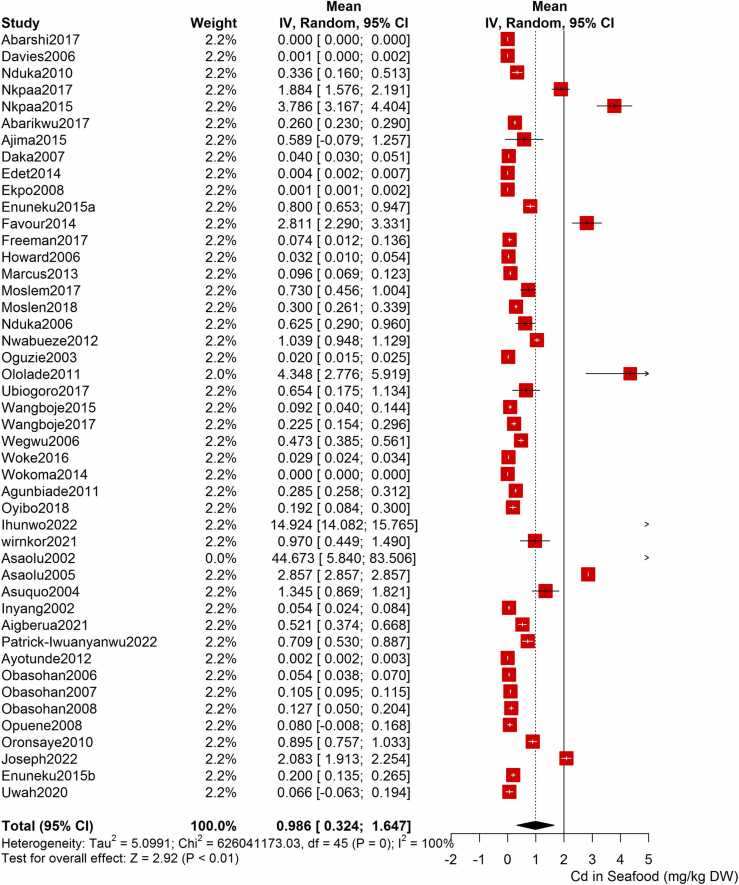
Forest plot representation of the meta-analysis of the Cd contents of seafood.

**Fig. 6:**
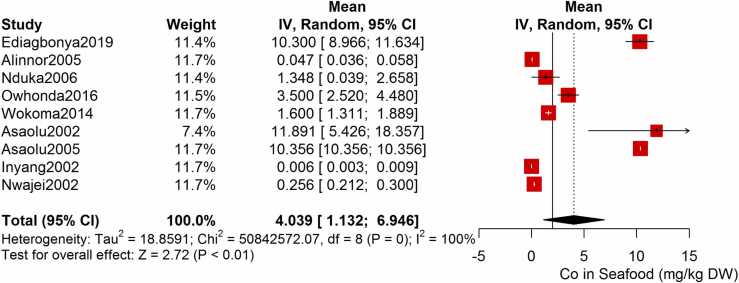
Forest plot representation of the meta-analysis of the Co contents of seafood.

**Fig. 7:**
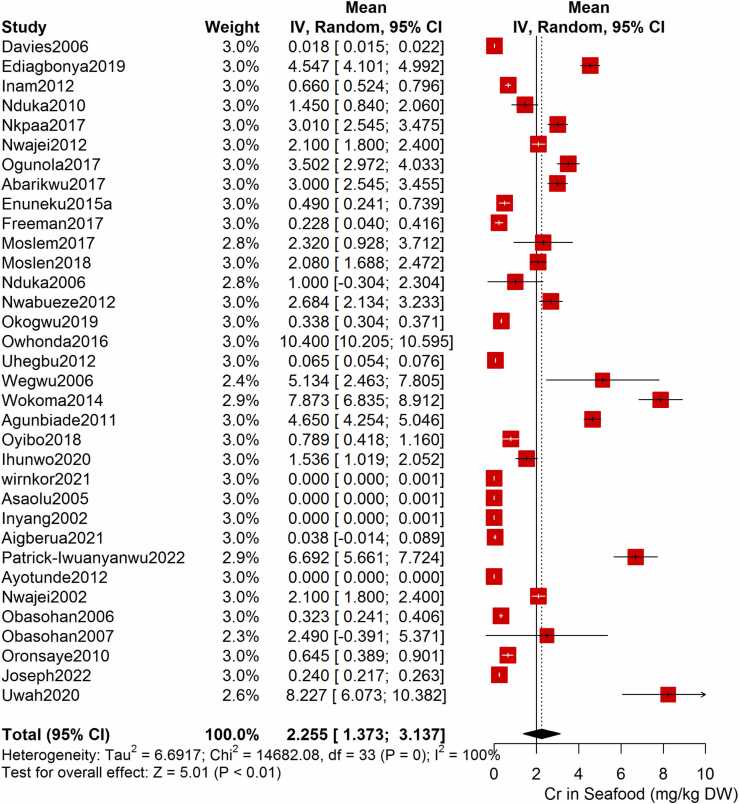
Forest plot representation of the meta-analysis of the Cr contents of seafood.

**Fig. 8:**
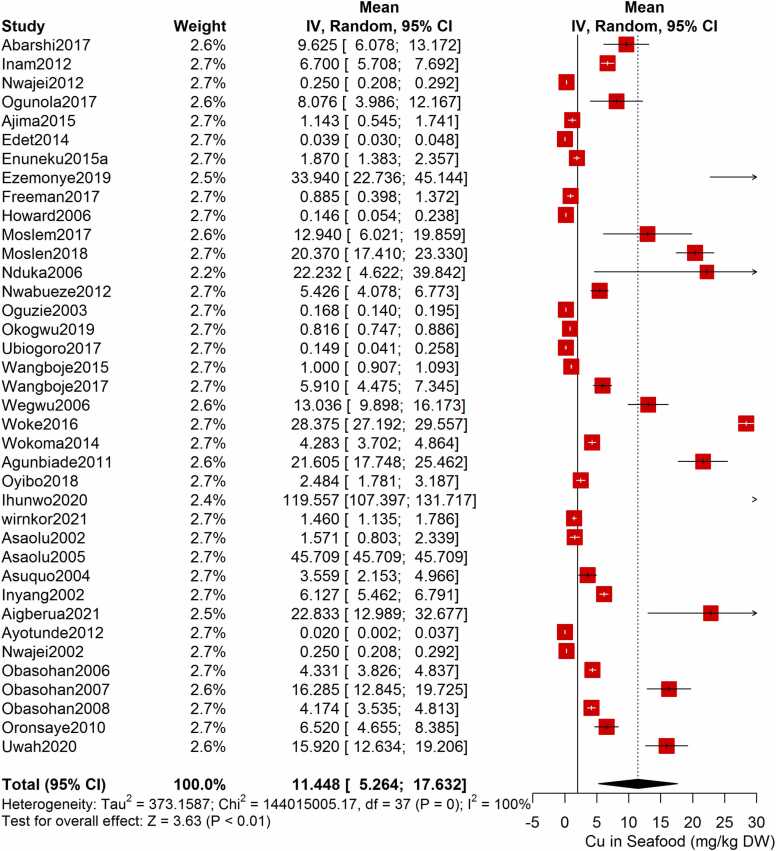
**:** Forest plot representation of the meta-analysis of the Cu contents of seafood.

**Fig. 9:**
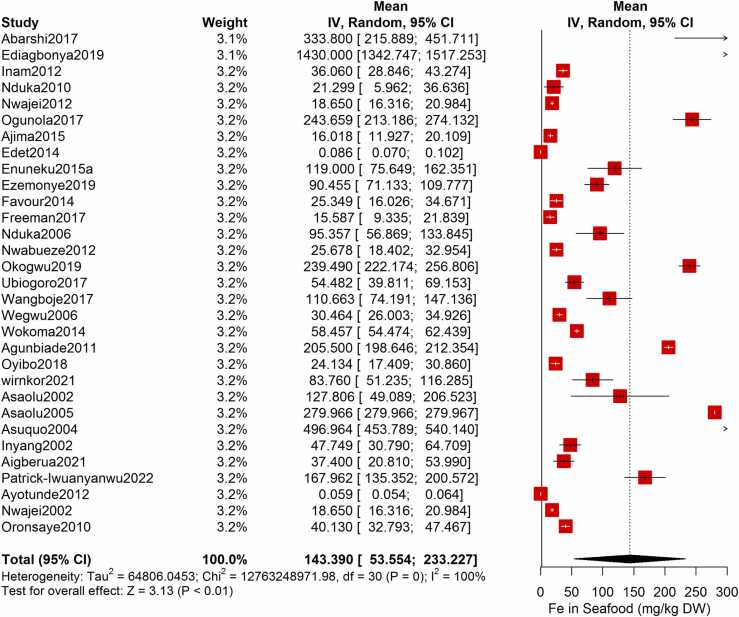
**:** Forest plot representation of the meta-analysis of the Fe contents of seafood.

**Fig. 10:**
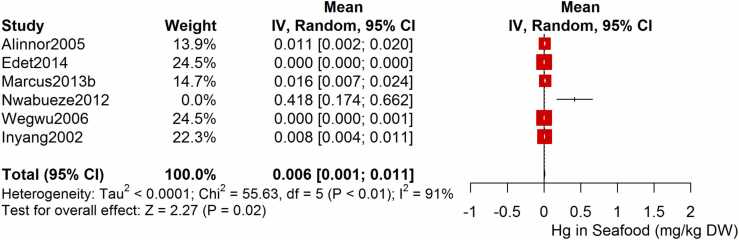
**:** Forest plot representation of the meta-analysis of the Hg contents of seafood.

**Fig. 11:**
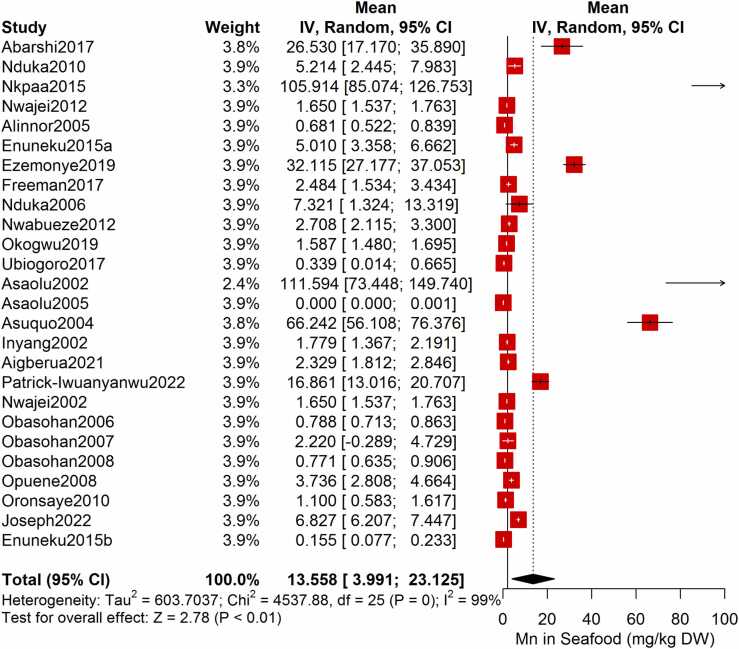
**:** Forest plot representation of the meta-analysis of the Mn contents of seafood.

**Fig. 12:**
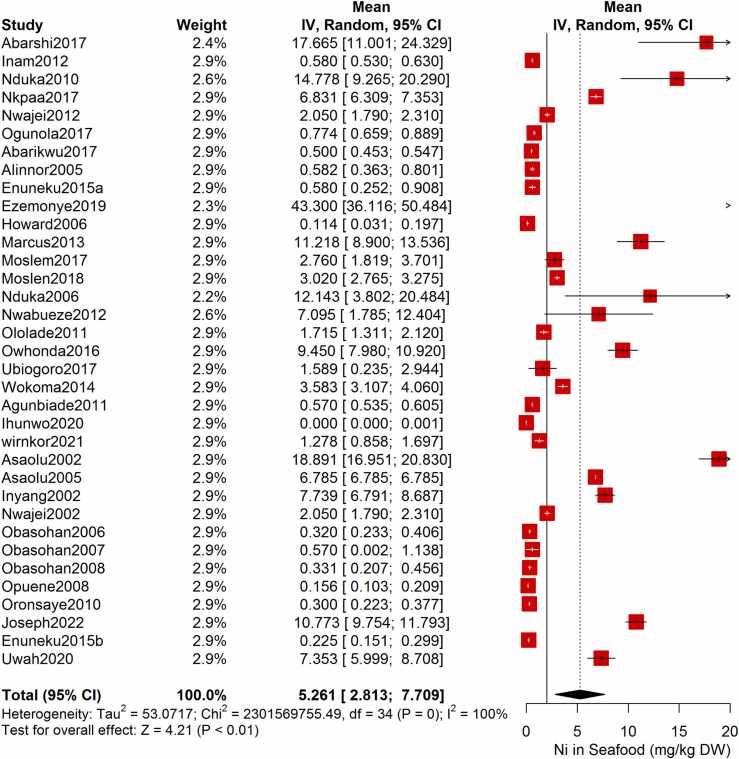
**:** Forest plot representation of the meta-analysis of the Ni contents of seafood.

**Fig. 13:**
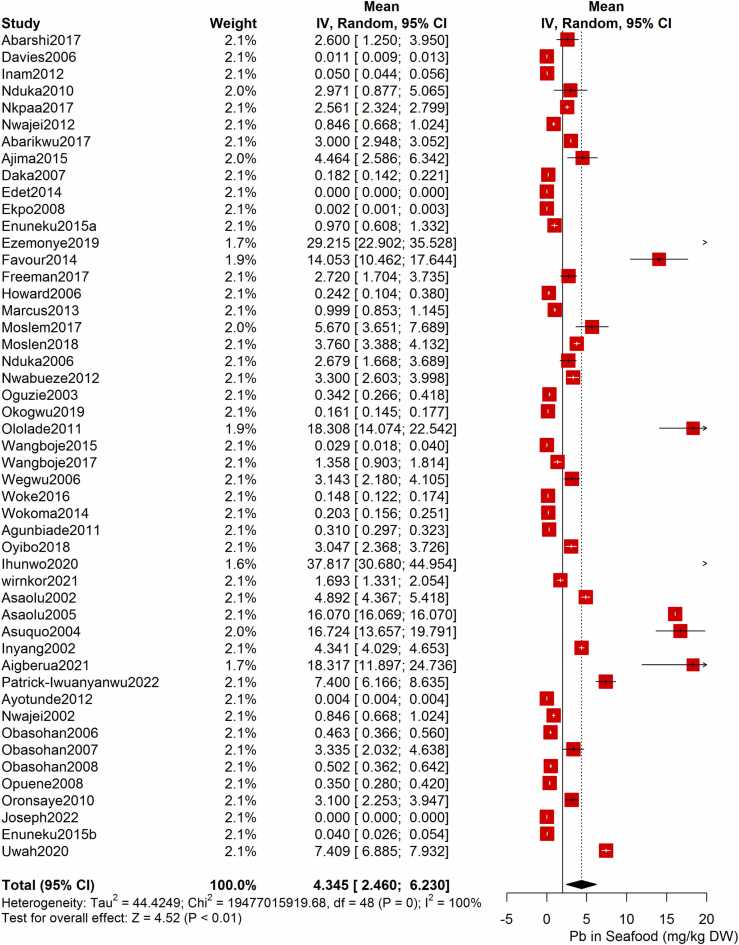
**:** Forest plot representation of the meta-analysis of the Pb contents of seafood.

**Fig. 14:**
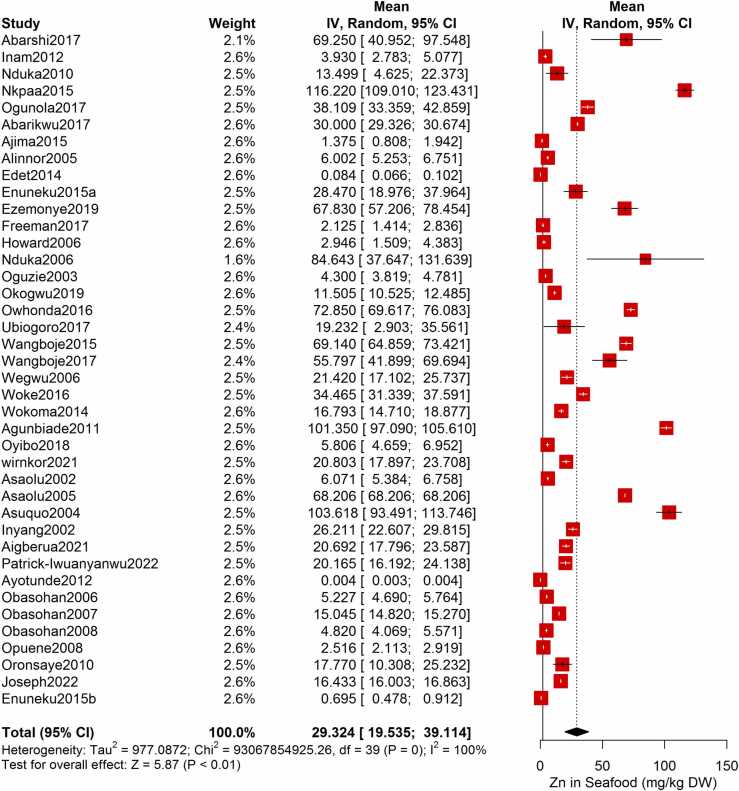
**:** Forest plot representation of the meta-analysis of the Zn contents of seafood.

Following the meta-analysis of the As contents of seafood derived from 10 studies and 243 seafood samples, a PME value of 0.777 mg/kg dry weight (DW) seafood (CI= 0.075; 1.479, I^2^ =100%) was obtained (Fig. 4). The PME computed for Cd from a total of 2481 seafood samples investigated across 46 studies was 0.986 mg/kg DW seafood (CI= 0.324; 1.647, I^2^ =100%) (Fig. 5) while Co had a PME of 4.039 mg/kg DW seafood (CI=1.132; 6.946, I^2^ =100%) computed from a total of 108 seafood samples investigated across 9 studies (Fig. 6). The PME for Cr, determined from 34 studies comprising a total of 1672 seafood samples is 2.255 mg/kg DW seafood (CI=1.373; 3.137; I^2^ =100%) (Fig. 7) while Cu had a PME of 11.448 mg/kg DW seafood (CI= 5.264; 17.632, I^2^ =100%) derived from 38 studies consisting of a total of 1743 seafood samples (Fig. 8). Fe had a PME of 143.390 (CI=53.554; 233.227; I^2^ =100%) from 31 studies consisting of 1109 seafood samples (Fig. 9) while Hg and Mn has a PME of 0.0058 mg/kg DW seafood (CI=0.0008; 0.0108, n = 6 studies) and 13.558 mg/kg DW seafood (CI= 3.991; 23.125, n = 26 studies) consisting of 116 and 1385 seafood samples respectively (Fig. 10, Fig. 11). The PME derived for Ni, Pb and Zn are 5.261 mg/kg DW seafood (CI=2.813; 7.709, n = 35 studies, seafood samples = 1827)(Fig. 12), 4.345 mg/kg DW seafood (CI=2.460; 6.230, n = 48 studies, seafood samples =2678) (Fig. 13), and 29.324 mg/kg DW seafood (CI=19.535; 39.114, n = 40 studies, seafood samples=2080) (Fig. 14) respectively.

### 4.5 Heterogeneity and subgroup meta-analysis

As indicated in Figs. 4 - 14, a high heterogeneity was observed in the PME of all the metals. Hg and Mn that has a I^2^ value of 91% and 99% respectively, while the other metals (i.e. As, Cd, Co, Cr, Cu, Fe, Ni, Zn and Pb) had I^2^ value of 100%. Subgroup meta-analysis based on the seafood category did not improve the I^2^ of any of the heavy metal investigated except for a slight improvement in the I^2^ of Hg. Similarly, the Chi square test for seafood category difference was not significant (p > 0.05) for any of the heavy metal investigated **(**Table S1-S11**).** The prevailing anthropogenic activities in each study area significantly influenced the between-study variation in the As, Co, Mn, Ni and Pb contents of seafood from the NDRN (p < 0.05). However, only slight improvement in the I^2^ values of As and Co were observed after performing a subgroup meta-analysis based on the anthropogenic activities. Subgroup meta-analysis based on the state-wise categorization of seafood sampling location showed slight reductions in the I^2^ values for Cd, Co, Cr, Cu, Fe, Hg, Ni, Zn and Pb. The test for subgroup difference showed that the state-wise categorization significantly influenced the between-study variation in the seafood contents of all the metals investigated (p < 0.05) except for Mn. The study-year subgroup meta-analysis did not improve heterogeneity in any of the heavy metals while the test for Chi square differences in the Pb, As, Co and Cr subgroup meta-analysis indicate significant differences (p < 0.05) in the PME of seafood across different study years (Table S1-S11).

## 5 Publication bias assessment

**Fig. 15:**
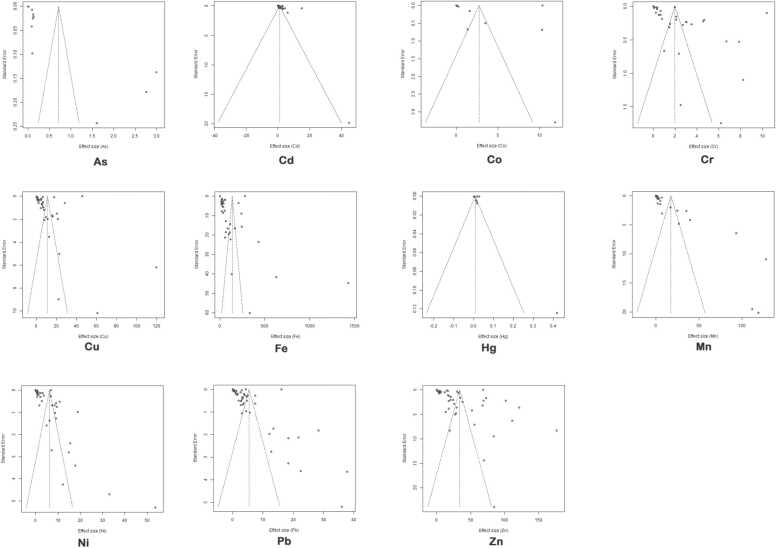
Funnel plot for the assessment of publication bias in the PME of each heavy metal. The p-value obtained from Egger’s test for each of the heavy metal is as follows: As (p = 0.003), Cd (p = 0.933), Co (p = 0.997), Cr (p = 0.001), Cu (0.0132), Fe (p = 0.2218), Hg (p = 0.9021), Mn (p = 0.000), Ni (p = 0.985), Pb (p = 0.882) and Zn (p = 0.636). Publication bias is confirmed when P-value from Egger’s test is < 0.05.

Outcome from the funnel plot indicates likelihood of publication bias in the dataset used for the meta-analysis of As, Cu, Cr, Fe, Pb, Mn, Zn and Ni. Of these, only those of As, Cr, Cu and Mn were confirmed by the Egger's test for bias at p < 0.05 (Fig. 15).

## 6 Sensitivity analysis

Sensitivity analyses identified 9 studies with outlying values for some of the heavy metals (Table 3). Upon exclusion of these studies from the meta-analysis, it was observed that they significantly influenced the pooled mean estimates (PME) of the affected metals, resulting in alterations ranging from 20% to 99%. Consequently, these studies were excluded from the meta-analysis.

**Table 3:** Outcome from the Sensitivity analysis showing the details of the highly influential studies excluded from meta-analysis.

Heavy metal	Study ID	Mean of study observations (95% CI)	PME (95% CI) before study exclusion	Percentage change in PME after study exclusion (%)	^##^ r-student statistics (mean of the r-student statistics of the remaining studies)	^##^ Cook.D Statistics. (mean of the CookD statistics of the remaining studies)
As	Ediagbonya2019	5.59 (4.79; 6.38)	1.20 (0.19; 2.22)	35.25	3.847 (−0.241)	0.669 (0.056)
Cd	Ihunwo2020	87(78; 97)	2.75(−0.35; 5.84)	64.21	16.29 (−0.118)	1.244 (0.002)
Co	Ezemonye2019	24 (15; 33)	5.59 (1.68; 9.50)	27.74	3.221 (−0.218)	0.608 (0.084)
*Cr*	Asaolu2002	32 (26; 39)	3.32 (1.67; 4.97)	18.67	6.065 (−0.087)	0.537 (0.021)
*Cr*	Nkpaa2015	18.67 (16.19; 21.16)	3.32 (1.67; 4.97)	15.70	3.765 (−0.021)	0.384 (0.026)
Cu	Ediagbonya2019	448 (420; 476)	22.56 (0.78; 44.34)	49.29	18.011 (−0.153)	0.999 (0.003)
Fe	Nkpaa2015	3479 (3109; 3849)	295.49 (69.07; 521.92)	31.52	7.162 (−0.124)	0.651 (0.014)
Fe	Nkpaa2017	2036 (1911; 2160)	295.49 (69.07; 521.92)	18.98	2.997 (0.006)	0.235 (0.027)
Hg	Wirnkor2021	35 (28; 41)	4.71 (−3.801; 13.22)	99.87	10.193 (−0.411)	1.173 (0.037)
Mn	Agunbiade2011	288 (278; 298)	31.79 (7.01; 56.56)	31.26	5.784 (−0.123)	0.007 (0.042)
Mn	Ediagbonya2019	204 (193; 216)	31.79 (7.01; 56.56)	20.44	2.986 (0.019)	0.264 (0.032)
Ni	Ezemonye2019	43 (36; 50)	5.20 (2.83 7.57)	20.32	6.495 (−0.103)	0.767 (0.016)
Pb	Nkpaa2015	62 (53; 71)	6.90 (3.24; 10.55)	18.1	5.033 (−0.03)	0.451 (0.019)
Pb	Ihunwo2022	83(67; 98)	6.90 (3.24; 10.55)	19.13	6.13 (−0.053)	0.494 (0.018)
Zn	Ediagbonya2019	383 (337; 429)	37.24 (19.88; 54.60)	21.26	8.954 (−0.126)	0.798 (0.009)

^##^ The exclusion of outlying studies from the meta-analysis generated a r-student statistics and Cook.D statistics that is much larger than what could be observed for non-outlying studies. The mean of the r-student statistics was computed for the other non-outlying studies and was placed in parenthesis for comparison sake. The mean of the Cook.D statistics was computed for the other non-outlying studies and was placed in parenthesis for comparison sake.

A repeat of the sensitivity analysis after excluding the outlying studies further identified studies by Nkpaa et al. [71], Ihunwo et al. [65], Owhonda et al. [26], Ediagbonya et al. [110], and Asaolu [60] as having the most influence on the PME computed for As (PME when excluded = 0.526), Cd (PME when excluded = 0.625), Cr (PME when excluded = 1.976), Fe (PME when excluded = 99.626), and Mn (PME when excluded = 10.880) respectively. The study by Ihunwo et al. [23] had high influence on the PME of Cu (PME when excluded = 8.482) and Pb (PME when excluded = 3.781) while the study by Ezemonye et al. [78] had the most influence on the PME computed for Ni (PME when excluded = 4.168) and Pb (PME when excluded = 3.937). The values reported by Nkpaa et al. [38] had obvious influence on the PME computed for Zn (PME when excluded = 26.943) and Mn (PME when excluded = 9.343).

### 6.1 Permissible limits in seafood

Result from this study indicated that the PME of As, Cd, Cr, Cu, Hg, Ni, and Zn are within the permissible levels recommended by various regulatory agency (Table 4) However, the PME obtained for Pb exceeded the 0.3 mg/kg limit recommended by FAO [116] by 14 folds.

**Table 4:** The pooled mean estimates (PME) of heavy metal in seafood from the Niger Delta Region of Nigeria (NDRN) in comparison to standard permissible limits in seafood.

Heavy metal	PME (mg/kg Seafood)	Permissible limits (mg/kg Seafood)	Reference
As	0.777	86	[117]
Cd	0.985	2	[116]
Co	4.039	-	
Cr	2.255	12	[117]
Cu	11.448	20	[118]
Fe	143.39	-	
Hg	0.0058	1.2–1.7	[116]
Mn	13.557	-	
Ni	5.261	80	[117]
Pb	4.35	0.3	[116]
Zn	29.324	50	[118]

Permissible levels for Fe, Co, and Mn were not specifically defined by FAO [116]. However, the PME obtained for Fe and Mn in the meta-analysis were found to be below the recommended tolerable upper intake levels of 45 mg/day and 6.3 mg/day, respectively, as indicated by Otten et al. [119]. These values were based on an assumption of a daily consumption of approximately 26.3 g of the seafood. The PME obtained for Co exceeded the EPA's provisional reference dose of 0.0003 mg Co/kg/day [43] under the assumption that a 60 kg adult consumes 26.3 g of the seafood.

### 6.2 Human health risk assessment

The EDI and THQ for each heavy metal are presented in Table 5. As shown in the table, Cd, Co and Pb have THQs of (8.981), (1.227), and (19.830) respectively while the other heavy metals had THQs lower than 0.2. According to the USEPA [43], the heavy metals As, Cr, and Pb have known potentials to cause cancer when ingested orally. In seafood from the NDRN, the estimated cancer risks associated with these metals are as follows: 1.06E-04 for As, 1.03E-04 for Cr, and 3.37E-06 for Pb.

**Table 5:** Non-carcinogenic health risk posed by the consumption of seafood obtained from the Niger Delta Region of Nigeria (NDRN).

	EDI (mg/kg/day)	THQ
As (inorganic)	7.08E-05	0.236
Cd	8.98E-05	8.980
Co	0.00038	1.227
Cr (III)	0.00021	0.00013
Cr (VI)	0.00021	0.0685
Cu	0.00104	0.0260
Fe	0.01307	0.0186
Hg	5.29E-07	0.0052
Mn	0.00123	0.0088
Ni	0.00048	0.0239
Pb	0.00041	19.830
Zn	0.00267	0.0089

THQ: Target Hazard Quotient, EDI: Estimated Daily Intake

## 7 Discussion

Seafood are important sources of protein, vitamins, essential minerals, essential fatty acids and income to rural dwellers in the NDRN [120], [121]. It is not only affordable but also easily accessible as it is harvested by peasant fishermen from the abundant marine resources in southern Nigeria [42]. This is of utmost significance given the high prevalence of malnutrition in Nigeria, where approximately 33% of children under the age of five suffer from stunted growth [42]. The importance of seafood is further highlighted by the prevailing rates of under nutrition and micronutrient deficiencies, particularly among the poor and rural populations [42]. According to Kingsley et al. [122], Nigeria experiences annual losses of over $1.5 billion due to vitamin and mineral deficiencies. For many households, especially those in rural and socioeconomically disadvantaged areas, seafood serves as a valuable and irreplaceable source of animal protein [122]. In fact, Kingsley et al. [122], estimates that seafood contributes over 25% of the Recommended Nutrient Intake (RNI) for Fe and Zn among pregnant and lactating women. Furthermore, fish-based nutritional strategy have been considered an important way to prevent childhood malnutrition in rural African countries [123].

While seafood is instrumental in providing a nutritious diet and combating deficiency diseases, the issue of contamination poses a significant challenge. The widespread pollution of several water bodies in the NDRN with heavy metals and other pollutants as a result of the intensity of the crude oil explorations and exploitations conducted in this region, as well as due to poor managements of industrial effluents and municipal wastes [4], predisposes marine species to possible contaminations by pollutants such as heavy metals. Such contaminations could endanger the survival and diversities of these aquatic animals as well as predispose human consumers to several health risks. The knowledge of the current status of elemental contamination of seafood from NDRN is therefore pertinent for safeguarding public health, preserving seafood biodiversity and for bio-monitoring environmental pollution in the NDRN. In this study, we have applied the systematic review and meta-analytic technique to review and compute PMEs of predominant heavy metals using data from studies that investigated the elemental contents of seafood samples obtained from natural water bodies in the NDRN.

Evidence from the included studies revealed a low to moderate level of heavy metals in seafood collected from the NDRN. However, few studies from this region reported very high levels of some heavy metals. For instance, unusual levels of Pb, Cr, Fe and Cd were observed in seafood obtained from Ogoniland and Woji Creeks. Both sites have been severely impacted by decades of crude oil pollution and suffers from various environmental deterioration [23], [65], [71], [38]. Similarly, hazardous levels of Pb, Ni and Co were detected by Ezemonye et al. [78] in seafood sampled from the Benin river. The presence of a bitumen industry near the river, coupled with the release of municipal and industrial wastes into the Benin river [79], may partly account for the unusual levels of these metals in the seafood. Wirnkor et al. [62], reported an alarming concentration of Hg in seafood sampled from Oguta Lake (Imo State). The environment of this lake is being impacted by intense crude oil exploration activities and crude oil spill incidents [62]. Previous studies have detected high levels of heavy metals in water bodies situated in regions with intense oil exploration activities [124], [125], [126], [127], [19]. Finding from such studies, which agrees with the present observation suggests a close relationship between oil exploratory activities and the contamination of the aquatic environment with heavy metals. High levels of Mn, Cr and Fe were seen in seafood obtained from some water bodies in Ondo State [110], [22], [60]. The high values of Mn and Fe observed in seafood from Oluwa river as reported by Ediagbonya et al. [110] is of significant interest since the environment around the study location is without obvious industrial activities. This may suggest a regional transfer of heavy metal pollutants from polluted upstream regions to downstream regions with less anthropogenic activities [128]. The high levels may also be due to other unknown natural or anthropogenic factors. Consequently, as a matter of public health emergency, fishing activities should be temporarily suspended in regions where hazardous levels of heavy metals were detected in seafood until appropriate agencies conduct a thorough investigation to ascertain the levels and actual sources of the heavy metals in both water and seafood from affected region.

The quality of the included study has a direct impact on the validity and usefulness of the PME derived from a meta-analysis [129]. Several tools such as Jadad Scale [130], the Newcastle-Ottawa Scale [131], the Risk of Bias Assessment Tool, the Cochrane Risk of Bias Tool [132], and the Quality Assessment Tool for Quantitative Studies [133] have been developed for the evaluation of the quality of studies used for meta-analysis and systematic review. The absence of an appropriate guideline for assessing the quality of environmental studies necessitated the adaptation of our previous method [4] as well as allotting scores for different study assessment criteria as is currently used in study-quality assessment tools like Newcastle-Ottawa Scale and Jadad Scale. The observation that the studies we omitted from meta-analysis due to insufficient details had an average score of 39.2% as against 62.7% for those included for meta-analysis, highlights the potential of this approach to provide an objective way of identifying or screening out low quality studies. The average score of 62% for studies used in the present meta-analysis suggests that the included studies were of moderate quality.

Meta-analysis provides a statistical method for combining the results from different studies in order to obtain a more precise estimate [134], [135]. Meta-analysis technique was applied in the present study to compute PME for each heavy metal using data reported in different studies. This technique is finding newer applications in the analysis of data from environmental studies [136], [137], [138], [139]. The PMEs of the heavy metals as computed in the present study showed some variations with the levels detected in seafood from other regions of the world. The values of As, Cu and Zn in our study were similar to those detected in seafoods from New Jersey, Ghana, Bolivia and Turkey but the values of Cr and Hg were lower compared to those from Spain, Bolvia and Ghana (Table 6). Our study found higher PME for Fe, Mn, Ni and Pb compared to those reported in several studies [141], [142], [140].

**Table 6:** Levels of heavy metals (in mg/kg wet weight) detected in muscles of seafood from around the world.

Study Location	As	Cd	Co	Cr	Cu	Fe	Hg	Mn	Ni	Pb	Zn	Reference
Niger Delta Region, Nigeria	0.08–1.48	0.32–1.65	1.13–6.95	1.37–3.14	5.26 − 7.63	53.55–233.22	0.001–0.011	3.99–23.12	2.81–7.70	2.46–6.23	19.55–39.11	Present study
Antalya, Turkey	-	-	0.33–0.86	0–0.57	1.03–5.11	3.25–8.12	-	0.09–0.28	-	-	1.31–4.09	[143]
Cheneb River, Pakistan	-	0.04–2	0.01–3.2	0.03 − 10	0.01–9	2–175	-	-	0.01–2	-	10.0–88	[144]
Bay of Algeciras, Spain	27–73	-	0.04–0.25	-	-	-	-	-	0.05–0.17	-	-	[145]
Canary Island, Spain	-	0.007	0.01	6.55	0.7	-	-	0.02	0.12	0.03	-	[142]
Marmara, Aegean and Mediterranean seas of Turkey	-	-	0.04–1.75	0.32–6.48	7.46–40.1	-	-	0.22–3.97	0.33–0.86	4.49–11.2	-	[146]
Bolivian Amazon, Bolivia	-	0.01–0.03	-	-	0.17–4.65	-	0.009–1.93	-	0.18	-	-	[147]
Canary Island, Spain	-	0.002–0.0021	0.0068–0.057	0.11–0.12	0.5–0.63	5.43 − 7.98	-	0.1–0.16	0.027–0.04	0.029–0.025	-	[140]
North west of Black Sea	7.725	0.433	BDL	BDL	4.07	40.34	-	-	BDL	BDL	66.09	[148]
New Jersey	0.23–3.30	0.0001–0.03	-	0.03–0.034	-	-	-	0.11–0.98	-	0.04–0.34	-	[12]
Ankobra, Ghana	0.03–0.12	0.02	0.05	0.05	-	-	0.35–1.15	0.02–0.8	0.01–0.04	0.02	0.60–0.71	[141]

The present study revealed that seafood from the NDRN is a rich source of essential metals and could contribute significantly to the recommended dietary allowances (RDA). Consumption of 26 g of this seafood on a daily basis can supply the RDA for Cu (0.3 – 1.3 mg) and Cr (0.005 – 0.045 mg). Additionally, the Mn, Zn, and Fe content in the seafood has the potential to fulfill 29%, 30%, and 53% respectively of the RDA for Mn (1.2–2 mg), Zn (2.5–12 mg), and Fe (7 – 27 mg) [119]. Essential elements such as Cr, Cu, Fe, Mn, and Zn play critical roles in the body as cofactors of enzymes involved in various metabolic processes. Their adequate dietary intake is crucial in preventing deficiency syndromes [119]. Mn is involved in amino acid, cholesterol and carbohydrate metabolism as well functions in bone development while Cr enhances the activities of insulin thereby assisting in glucose homeostasis [119]. Cu protects against oxidative stress and inflammation through its involvement in the activities of oxidases. Fe, as constituent of heme, facilitates the transport of O_2_ across the body while Zn functions as cofactors in proteins involved in gene expression and developmental processes [119]. Deficiency of Mn, Cr, Cu, Fe and Zn have been associated with scaly dermatitis, increased insulin requirements, osteoporosis, anemia and impaired immunity respectively [119].

The THQ, which is a ratio of the EDI of the contaminant of interest to a standard reference dose (i.e. the maximum exposure dose of each contaminant that is not expected to elicit any adverse effect) gives a value that indicates the extent to which the ingested dose exceeds the maximum safe levels of the contaminant in question [149]. THQs of less than 1 is not considered to elicit a non-carcinogenic effect whereas THQ greater than 1 may trigger an observable non-carcinogenic health risk to persons exposed to the contaminants [150]. The THQs derived from this study indicated that chronic consumption of seafood from the NDRN poses significant non-carcinogenic risks to inhabitants of the region. These risks were majorly due to the, Cd, Pb and Co contents of the seafood which has THQ of 8.98, 19.83 and 1.23 respectively.

Exposure to Cd have been reported to weaken the skeletal system by increasing the demineralization of bones, reducing bone density therefore increasing the risks of bone fractures and osteoporosis [14], [151]. Cd-exposure also impairs cognitive and kidney developments in infants as well as exert liver, kidney, reproductive and cardiovascular toxicities [14]. Cd-induced renal damage is characterized by proteinuria, tubular lesion and Ca loss while its anti-reproductive effect is characterized by reduced sperm count, reduced sperm quality and increased incidence of abortion [151].

Cobalt is an important constituent of cobalamin (i.e. Vitamin B12) which is produced in some animals through the activities of intestinal microorganism [152]. Co is required only in minute quantities (i.e. 0.1 – 2.4 µg/day) and is essential in association with vitamin B12, for normal blood formation and neurological functions [152], [119]. Deficiency of dietary Co (in forms of cobalamin) have been associated with hematological, neurological and gastrointestinal defects [119]. On the other hand, evidence from human and animal studies showed that high oral intake of Co could exert various toxic effects such as Polycythemia (i.e. excess production of red blood cells) and adverse thyroid and cardiac effects [153]. Exposure to Co have been reported to alter neurological behaviors and trigger neurodegeneration in animals [154]. The neurodegenerative effects of Co is thought to be mediated by the activation of hypoxia-inducible factor-1α pathway leading to increased reactive oxygen species generation, defective autophagy and accumulation of toxic proteins [154]. Oral exposure to cobalt at doses of 0.0007 mg/kg/day is known to flare up dermatitis in humans [153]. A recent epidemiological study associated urinary Co with increased risk for obesity, insulin resistance, metabolic syndrome and elevated triglycerides [155]. The authors concluded that there was no safe threshold below which Co did not exert an adverse effect [155]. The EPA provisional RfD of 0.0003 mg Co/kg/day reported in the latest [43] release, was derived from the potentials of Co to decrease the uptake of iodine in human [153].

Pb is a known systemic toxicant that affects several organs including the kidney, bone, brain, and the reproductive organs [15]. Human studies have shown that occupational exposure to Pb reduced sperm count, sperm quality and chances of fathering a child [156], [157]. In females, Pb exposure alters the production of hormones, reduces fertility potentials, impairs menstruation, delays conception time and triggers other adverse pregnancy outcomes [158], [159]. The reprotoxic effect of Pb has been linked with its potential to disrupt the endocrine system, trigger oxidative stress and interfere with gene expression [160], [161], [156]. Pb also interferes with the development of the nervous system in infants leading to life-long impairment in the cognitive abilities of exposed children [162], [44]. The current standpoint is that Pb, at every level of exposure, can trigger an observable effect especially amongst the most vulnerable groups especially the infants and fetuses of pregnant women [44]. Due to the absence of an RfD value for Pb in the latest [43] listings, we used the United States Food and Drug Administration (FDA) interim reference level (IRL) of 8.8 µg Pb/day (approximated as 0.000146 mg/kg bw/day for a 60 kg female) currently established for females of childbearing age [44] for estimating the health risk. It was estimated that at this exposure level (i.e 8.8 µg Pb/day) the blood Pb level would not exceed 0.35 µg/dL or cause up to 1 point decrease in the intelligence quotient of the exposed infants [44].

According to the USEPA [43], inorganic As, Cr (VI), and Pb PO_4_ can trigger cancer when inhaled or ingested whereas Cd, Co and Ni exposure have been documented to trigger carcinogenesis following inhalation but not through oral exposure [43]. Since seafood are majorly ingested we have considered only the potential cancer risk due to oral exposure, however this does not rule out the possibility of carcinogenic risks due to inhalational exposure, for instance amongst workers in industries that convert seafood to powdered products. According to our data, ingestion of seafood from the NDRN pose considerable cancer risk due to the As, and Cr contents of the seafood. As is a class A human carcinogen that has been reported to increase the incidence of skin, kidney, bladder and lung cancers in humans [163], [164], [165]. Cr (III) and Cr (VI) are the most stable oxidative states of Cr in nature with Cr (III) being less toxic and less permeable to living tissues than the Cr (VI) oxidative state [166]. Both forms of Cr have been detected in seafood [167] and are in fact inter-convertible. For instance, Cr (VI) is readily reduced to Cr (III) in living tissues, while Cr (III) is converted to Cr (V1) under certain environmental and experimental conditions [168]. Occupational exposure to Cr (VI) compounds have been reported to trigger the cancer of the lung, nose and nasal sinus. While limited evidence exists on its potential to cause stomach cancer in man, animal study showed increased incidence of UV-induced skin tumor following oral ingestion of Cr (VI) [169]. The cancer slope factor of As was derived from exposure level that could trigger skin cancer in humans [170] while that of Cr (VI) was derived from the exposure concentration that is likely to trigger the cancer of the gastrointestinal tract in humans [171]. Pb salts, particularly those of phosphates and acetates, is known to trigger renal tumors in animals especially when exposed to high doses of the salt [172]. Pb compounds are currently classified as probable human carcinogen Class B2- [172]. Accumulated evidences indicates that Pb may acts synergistically with other environmental pollutants to increase the incidence of cancer through their abilities to modulate gene expressions [173].

The life time cancer risk gives a numeric indication of how many new cases of cancer can result within a population of people who have been similarly exposed to a particular agent [174]. The values of 1.06E-04 and 1.03E-04 derived for As and Cr respectively, implies that one additional case of cancer could occur for every 10,000 consumers of seafood due to the As or Cr contents of the seafood while the value derived for Pb (i.e. 3.44E-06) implies that three additional case of cancer could result amongst a million seafood consumers. The Agency for Toxic Substances and Disease Registry (ATSDR) considers cancer risk values above 1E-04 to be of concern for an increased risk of cancer in a human population [174]. This suggests that the consumption of seafood from the NDRN predisposes consumers to increased risk of the carcinogenic effects of As and Cr.

The I^2^ test for heterogeneity assesses the presence and extent of inter-study differences between studies combined for meta-analysis [175]. I^2^ values less than 40 is considered low heterogeneity while 30–60% and above 75% is considered moderate and high heterogeneity respectively [175]. The high heterogeneity observed in the present study is similar to previous observations made when meta-analysis was performed on environmental data [137], [139], [176]. Even though some of the subgroup categories (such as anthropogenic activities, state-wise and study year categories) exerted significant influences on the metal content of seafood, the lack of improvement in the I^2^ values after subgroup meta-analysis, showed that none of the subgroup category could fully explain the sources of the observed heterogeneity in the PME of the heavy metals. This indicates that the heterogeneity observed in this study is likely influenced by multiple factors, rather than being solely attributed to a single factor. It also implies that additional factors beyond the ones investigated in this study needs to be considered for better understanding of the sources of the observed variations. Other potential sources of heterogeneity not accounted for in this study includes seasonal variation [128], methodological differences [177], seafood specie-specific differences as well as differences due to seafood feeding habits, sizes or gender [178], [148], [179], [149].

## 8 Limitation

The strength of this study lies in the numbers of studies used in the computation of the PME, this gave a more precise data than those from the individual studies. An additional strength is the rigorous quality assessment we conducted for the included studies as well as the at-a-glance overview of the study quality we provided. Despite our effort to retrieve all relevant paper in this subject area we acknowledge the possibility of unintentional omission of some relevant studies either due to the keywords we used or the literature databases we searched. Since this study relied solely on the data from other studies, the validity, usefulness and reliability of the PMEs and risk assessments are subject to the quality and authenticity of each included studies. For instance, as seen from the quality assessment section of this paper, majority of the study did not provide evidence that equipment or method validation was carried out before elemental analysis. Similarly most of the included study had high likelihood of assessment and sampling bias while a few of the studies had high risk of publication biases. The inherent biases and limitations of the included studies could limit the accuracy of the PMEs and the conclusions drawn about the potential health risks to inhabitants of the NDRN. The high heterogeneity in the meta-analysis might impair the generalizability of the PME (of each heavy metal) for the entire NDRN’s population, however, the ease of trans-border trades amongst communities of the NDRN predisposes majority of the inhabitants to an average exposure level that may be close to the computed PMEs. This assertion is supported by evidence from the sensitivity analyses which showed that the PME of most of the metals was robust enough to prevent a drastic change in its value following the removal of any particular study. The presence of uneven group sizes and low number of studies within each subgroup (in some cases, one study), and high heterogeneity in the dataset may potentially affect the outcomes and conclusions drawn from the subgroup analyses [180].

## 9 Conclusion and recommendation

This systematic review and meta-analysis was necessitated by the need to comprehensively evaluate the metal contents of seafood in the NDRN and determine their potential health risks. The findings of this study raise serious concerns regarding the safety of seafood from the NDRN. Not only do the Pb levels exceed thresholds associated with decreased intelligence quotient in children, but the Cd and Co contents also surpass safe limits for renal and thyroid dysfunctions respectively. Additionally, the presence of As and Cr in seafood has potentials to increase the risk of skin and gastrointestinal cancers among consumers. These alarming results have significant implications for public health and economic growth of the region since majority of the inhabitants rely heavily on seafood as their primary source of essential nutrients.

To mitigate these potential adverse effects, consumers are encouraged to minimize seafood consumption and to diversify their sources of animal proteins to include non-seafood options. This would reduce the accumulation of specific heavy metals to levels that can cause adverse effect. To ensure a sustainable and healthy seafood supply in the NDRN, we recommend that relevant regulatory agencies should conduct frequent monitoring of seafood from this region especially in locations such as Ogoniland (Rivers State), Woji Creek (Rivers State), Benin River (Edo State) and Oguta lake (Imo State) where unusual levels of toxic metals were detected. Such monitoring activities should focus at identifying point sources of marine pollution as well as restricting fishing at highly contaminated regions. Stricter enforcements of regulations against the release of industrial effluents, municipal wastes and crude oil related substances into water bodies are equally recommended.

Finally, in order to improve the quality of future bio-monitoring data generated from the NDRN, appropriate method validation should be performed prior to sample analysis and efforts should be made to reduce sampling and detection biases.

## Ethics approval

Not Applicable.

## Funding

The authors did not receive support from any organization for the submitted work.

## CRediT authorship contribution statement

**Francis Uchenna Umeoguaju:** Conceptualization, Methodology, Formal analysis, Investigation, Resources, Data curation, Writing – original draft. **Joyce Oronne Akaninwor, Eka Bassey Essien and Benjamin Achor Amadi:** Supervision, Writing – review and editing. **Chimaobi James Ononamadu:** Validation, Investigation, Resources, Data curation. **Onyedika Igboekwe and Charles German Ikimi:** Validation, Data curation. All the authors read and approved the manuscript.

## Declaration of Competing Interest

The authors declare that they have no known competing financial interests or personal relationships that could have appeared to influence the work reported in this paper.

## Data Availability

The data used for this study can be obtained from the corresponding author upon reasonable request.
