# Supplementary material for: Heavy metals contamination of seafood from the crude oil-impacted Niger Delta Region of Nigeria: A systematic review and meta-analysis
Source: Toxicol Rep. 2023 Jun 20;11:58–82. doi: 10.1016/j.toxrep.2023.06.011 (PMC10320387; doi:10.1016/j.toxrep.2023.06.011)
Supplement: Supplementary file 1 — Supplementary material. [file mmc1.docx]

**Table 1S:** Sampling coordinates anthropogenic activities and seafood reported in included studies.

| **Author & year** | **Sampling coordinates** | **Anthropogenic Activities at Sampling site** | **Seafood types** |
| --- | --- | --- | --- |
| Ataikiru2008 | - | Presence of crude oil pipelines, Tank farm reservoir and crude oil loading activities to oil tankers. | *Periwinkles and Shrimps* |
| Omobepade2020 | Lat (6^o^00' - 6^o^30'N), Long (4^o^45' - 5^o^45’E) | Fishing, farming and other domestic activities. | *Nematopalaemon hastatus* |
| Ighariemu2018 | Lat (5°00- 6°.30′N), Long (5°00 - 6°.45′E) | Farming, fishing, and network of crude oil pipelines. | *Chrysichthys nigrodigitatus, Clarias gariepinus, Schilbe mystus* |
| Ibanga2021 | Lat (4^o^73.22’- 4^o^82.72’N), Long (7^o^04.40’ - ^o^07.49’E) | Effluents from residential, industrial and agricultural sources empties into the Woji Creek | *Uca tangeri* |
| Uwah2020 | Lat (4°45′- 5°00′ N), Long (6°50′- 7°00′E). | Domestic, agricultural, industrial and commercial activities near water body. | *Tympanotonus fuscatus, Callinectes amnicola* |
| Enuneku2017 | Lat (5^o^58 - 6^o^03N), Long (5^o^24 -5^o^30E) | Transportation of crude oil product, fishing and domestic activities. | *Pomadasys peroteti, Albula vulpes,* |
| Joseph2022 | Lat (4^o^30’- 5^o^1'30N), Long (7^o^48' - 8^o^18'E) | Domestic, commercial and oil exploration activities. Some sampling regions had history of oil spill incidence. | *C. senegalensis* |
| Udo2012 | - | - | *Heterobranchus longifilis, Clarias gariepinus, Chrysichthys nigrodigitatus* |
| Oronsaye2010 | Lat (6^o^5’N Long (5^o^8’E | - | *Mormyrops deliciosus, Mormyrus*  *macrophthalmus* |
| Opuene2008 | Lat (5^O^01 - 5^O^05'N), Long (6^O^17’- 6^O^21'E) | Presence of flow stations, crude oil exploration activities. | *Macrobrachium felicinum* |
| Obasohan2008 | Lat (6^o^50’N), Long (5^o^80’E) | Municipal wastes disposal and domestic activities around water body. | *Erpetoichthys calabaricus* |
| Obasohan2006 | - | Water body receives municipal wastes, agricultural run-offs and effluents from wood treatment and rubber processing factory. | *Malapterurus electricus, Chrysichthys nigrodigitatus* |
| Nwajei2002 | - | Sampling site is in close vicinity of Delta Glass factory. | *Synodentis membraneceus* |
| Ayotunde2012 | Lat (4^o^25’ - 7^o^.00´N),  Long (7^o^15´ - 9^o^30´ E). | Water transportation and agricultural activities. | *Chryshchythys nigrogitatus*), |
| Inyang2002 | - | Sampling sites had previous history of oil spill incidence | *Chloroscombrus chrysurus,Chrysichthys*  *nigrodigitatus, Ethmalosa fimbriata, Ilisha africana, Sardinella maderensis,Pomadasys jubelini, Liza grandisquamis, Polydactylus quadrifilis, Pseudotolithus elongates, Tilapia guineenisis* |
| Eboh2006 | - | - | *Chsysichthyes nigrodigitatus, Oreochromis niloticus, Ilisha africana, Ethmalosa fimbriata, Periophthalamus koelreuteri* |
| Asuquo2004 | Lat (4^o^00 ' - 8◦00' N),  Long (7^o^20' – 10^o^00' E) | - | *Chrysichthys nigrodigitatus, Pseudotolithus elongatus, Tympanotonus sp., Portonus sp., Oreochromis niloticus, Ethmalosa fimbriata* |
| Asaolu2002 | - | - | *Oreochromis niloticus, Synodonthis* sp., *Clarias gariepinus* |
| Asaolu2005 | Lat (5^o^45’ - 6^o^30’N), Long (4^o^30’- 5'00"E) | - | *Oreochromis niloticus, Synodonthis* sp., *Clarias gariepinus* |
| wirnkor2021 | Lat (5^o^42’24”N), Long (6^o^47’33”E) | Sampling region is near to Oguta Oil field and had history of oil spill. | *O. leucostictus, H.fossilis, M. salmoides, C. spectaculurus* |
| Patrick-Iwuanyanwu2022 | Lat (4º42’7.31”N), Long (7 º28’13.71” E) | Crude oil related activities; the region had history of crude oil spill. | *E. Bostrichus., L. Lutjanuu., P. Polynemus., B. Cristictis., C. Ethomolussa., P. Pomadasys., C. Caranx., S. Scumbrius., S. Sinoglosis., B. Citharicthys* |
| Aigberua2021 | - | Brackish/marine habitat, Crude oil related activities | *Sarotherodon melanotheron, Chrysichthys*  *nigrodigitatus* |
| Ihunwo2022 | Lat (4°48’27.47″ - 4°49’33.10″N),  Long (7°2’39.83″ - 7° 3’38.40″E) | Water transportation, Urban residential areas, dump sites and industrial activities. | *Sarotherodon melanotheron* |
| Ihunwo2020 | Lat (7^o^1.80’ - 7^o^4.80’N),  Long (4^o^48.00' - 4^o^50.40'E) | Fishing and transportation of petroleum product and other commercial activities. | *Mugil cephalus* |
| Oyibo2018 | - | Active oil exploration in close vicinity of sampling sites. | *Cololabis saira, Mugil cephalus, Tylosurus crocodilus, Trichiurus lepturus, Oreochromis niloticus,Scomber scrombru, I. africana africana* |
| Agunbiade2011 | Lat (5◦50' – 6◦09'N),  Long (4◦45' – 5◦05'E) | Active oil exploration near sampling site. | *O. mossambicus, H. forskahlii* |
| Okoro2007 | Lat (5^o^23' - 5^o^26'N), Long (5^o^16' -5^o^22'E) | Active oil exploration at close proximity to river. | *Ethmalosa fimbriata* |
| Ossai2014 | - | Anthropogenic wastes empties into water body. | *Tilapia mariae, Clarias gariepinus* |
| Omuku2008 | - | - | *Tilapia zilli,*  *synodontis membraneceus* |
| Olowoyo2010 | Lat (5^o^00 -6^o^00N), Long (5^o^10 - 6^o^00E) | - | Catfish, Blue crab, crayfish |
| Ogunola2017 | Lat (4^o^44'00 -4^o^46'10"N),  Long (7^o^5'15"-7^o^6'15 E) | Receives effluents from Port Harcourt refinery as well as from municipal and industrial wastes. Active oil bunkering activities. | *Sarotherodon melanotheron, Chrysichthys nigrodigitatus* |
| Nkpaa2016 | Lat (4° 40′5″ - 4° 43′ 19.5″ N), Long (7° 22′ 53.7″ - 7° 27′9.8″E) | Presence of oil related industrial activities. Sampling area had history of crude oil spillage | *Tilapia guineensis, Liza falcipinis, Callinectes pallidus, Penaeus notialis* |
| Nkpaa2017 | Lat (4°40′5′′N - 4°43′19.5′′N ), Long (7°22′53.7′′ - 7°27′9.8′′E) | Presence of oil related industrial activities. Sampling area has history of crude oil spillage | *Callinectes pallidus, Tympanotonus*  *fucastus, Uca tangeri, Crassostrea gasar* |
| Inam2012 | Lat (4^o^30 - 4^o^45N), Long (7^o^30 - 8^o^45E) | Water body receives effluents from crude oil activities, industries and municipal wastes. | *Crassostrea rhizophorae* |
| Godwin2011 | Lat (4°39'18.460" -4°56'00.000"N), Long (5°48'43.052" -6°16'30.000"E) | Oil exploration and urban settlement around sampling sites. Presence of dump sites and industrial discharges near water bodies. | *Tilapia nicolitica sp,* |
| Edem2009 |  | Receives industrial and municipal waste effluents. | *Oreochromis niloticus* |
| Davies2006 | Lat (4^0^ 25' - 4^o^ 45' N),  Long (7^o^ 00' - 7^o^ 15' E) | Receives municipal wastes from Port Harcourt. Presence of abattoir, dredging company, and crude oil industries near the river. | *Tympanotonus fuscatus* |
| Abarshi2017 | Lat (4^o^26’0”), Long (7^o^10’0”) | Transport and agricultural activities.  Crude oil and non-crude oil industrial activities around water bodies. | *Genyonemus lineatus* |
| Marcus2013b | Lat (04^o^ 40' - 05^o^ 00'N), Long (07^o^ 00' - 07^o^ 15'E) | Receives effluent discharges from industrial and domestic sources. Presence of refinery and oil prospecting industries around sampling site | *Pachymelania aurita, Crassostrea rhizophorea, Periophthalmuskoelreuteri, Mugil cephalus, Sardinella marderensis, Tilapia guineensis* |
| Ideriah2006 | Lat (4^o^27′ 16″ - 4^o^54′N),  Long (6^o^56′04″ -7^o^ 35′ 27″ E) | Presence of petrochemical industries, oil prospecting and refineries around river body. Bonny river also receives agricultural and municipal effluents. | *Tympanotonus Fuscatus, Pachymelania aurita* |
| Freeman2017 | Lat (5°3’5.11”N), Long (  5°40’44.11”E) | Oil exploration and refinery industries located near sampling sites. River also receives municipal wastes | *Tympanostomus Spp, Callinectes amnicola* |
| Ezemonye2019 | Lat (05^o^59’43.6’’ -05^o^59’35.7’’N), Long (05^o^28’06.7’’- 05^o^25’56.2’’E) | Transportation and presence of multiple oil prospecting industries. | *Macrobrachium macrobrachion, Brycinus longipinnis* |
| Ezemonye2016 | Lat (05^o^59’43.6’’ – 05^o^59’35.7’’N), Long (05^o^28’06.7’’- 05^o^25’56.2’’E) | River receives inputs from municipal and industrial waste. Also bitumen processing plant along river stretch. | *Macrobrachium macrobrachion, Brycinus longipinnis* |
| Etesin2007 | Lat (4^o^25 - 4^o^45N), Long (7^o^19 - 7^o^45E) | Receives waste input from agricultural, urban, industrial and crude oil related activities. | *Ethmalosa fimbriata, Tilapia guineensis* |
| Chindah2009 | Lat (4^o^25" - 4^o^0 40" N),  Long (7^o^ 25" - 7^o^15" E) | Presence of flow stations, gas processing plants, crude oil tank farms near water source. Industrial and municipal waste discharges into water bodies | *Tympanotonus fuscastus* |
| Adebayo-Tayo2010 | - | - | *Penaeus Sp., Callinectes Sp* |
| Woke2016 | Lat (4° 34.18ʹN), Long (7° 22.10ʹE) | Fishing activities. Crude oil exploration activities near river. | *Crassostrea gasar* |
| Ubiogoro2017 | Lat ( 5°00 - 6°30' N), Long (5°00 - 6°45'E) | Oil producing community of Delta state | *-* |
| Owhonda2016 | - | Domestic, fishing and oil exploration activities around river. | *Tilapia guineensis, Sarotherondon*  *melanotheron* |
| Otitoju2013 | - | - | *Tympanotonus fuscastus* |
| Olowoyo2011 | Lat (5^o^00 - 6^o^00N),  Long (5^O^10 - 6^O^30E) | Intense oil exploration activities | Periwinkle, Tilapia |
| Nwoko2015 | Lat (04° 53’19.020”),  Long (06°53’53.086”E) | Presence of abattoir, poultry, fabrication companies, market, oil exploration, urban activities, loading and offloading of oil near river. | *Clarias gariepinus, Oreochromis niloticus* |
| Favour2014 | Lat (4^0^53” N), Long (6^0^ 55’’ E) | Presence of petrochemical industries and other urban activities around Iwofe river bank. | *Epinephelus aeneus, Lobotes surinamensis,Lutjanus goreensis,Callinectes amnicola,Chrysichthys nigroditatus* |
| Wokoma2014 | Lat (04^0^43’ - 04^0^37’N),  Long (06^0^46’ - 6^o^48’E) | - | *Pseudotolithus elongatus, Chrisichthys nigrodigitatus, Mugil cephalus* |
| Wegwu2006 | - | Water body receives effluents from petrochemical industries, refineries, agricultural activities and municipal wastes. | *Clarias gariepinus* |
| Wangboje2017 | Lat (5°43' - 5°30'N),  Long (6°20' - 6°12'E) | Water body receives effluent from domestic and market wastes. | *Brycinus intermidus, Clarias gariepinus,*  *Parachanna obscura, Ctenopoma kingsleyae, Hemichromis bimaculatus, Phractolaemus ansorgeii* |
| Wangboje2015 | Lat (7.106^o^N), Long (6.696^o^E) | Commerce, fishing, farming and mining activities near river | *Clarias gariepinus, Hemichromis fasciatus, Barbus occidentalis, Synodontis sorex, Alestes baremose, Chrysichthys nigrodigitatus, Oreochromis niloticus* |
| Uhegbu2012 | - | Oil exploration activities around sampling area | *Claria garie pinus, Crago nigricando, Palaemon serratus, Uca pugrlater,Scyll serrata, Anguilla anguilla, Litorina littorea, Harengula jaguana, Tilapia zilli* |
| Olusola2015 | - | Fishing, agricultural and recreational activities near water source. | Arius latisculatus, Cynoglossus browni, Caranx lugubris, Sardinella aurita, Caranx senegallus |
| Ololade2011 | Lat (5^o^45 - 6^o^30N),  Long (4^o^30 -5^o^00E) | Fishing, commercial and recreational activities takes place around the sampling location. | *Tilapia zilli, Callinectes sapidus,*  *Littorina littorea* |
| Okogwu2019 | Lat (5°47′ - 5°52′N),  Long (7°56′ - 8°01′E) | Mining activities along river basin. | *Chrysichthys nigrodigitatus,Clarias anguillaris, Tilapia zillii, Mormyrus rume* |
| Oguzie2003 | - | Presence of rubber factory, breweries, abattoir near river. | *Clarias gariepinus, Channa obscura,*  *Chromidotilapia guentheri* |
| Nwabueze2010b | - | - | *Egeria radiata* |
| Nduka2006 | - | Sampling region has a long history of oil spillage. | *Tilapia niloticus, Gynchus niloticus, Chrysichthys auratus, Ethmaliosa timbriata* |
| Moslem2018 | - | Receives domestic & industrial wastes. Dredging activity is ongoing near study area. | *Penaeus sp* |
| Moslen2017 | Lat (4^o^48' - 4^o^48'N),  Long (7^o^3'0 -7^o^3'43'E) | Receives domestic, municipal and industrial waste discharges. | *Callinectis amnicola* |
| Marcus2013 | Lat (04^o^40 – 05^o^00N),  Long (07^o^00 – 07^o^15E). | Bonny River receives effluent from Port Harcourt refining company through nearby Ekerekana Creeks. | *Pachymelania aurita, Crassostrea rhizophorae, Periophthalmus koelreuteri, Mugil cephalus, Sardinella Maderensis, Tilapia guineensis* |
| Howard2006 | - | Presence of oil well head around sampling station. Also receives input from runoffs of domestic wastes. | *Periophthalmus papillio* |
| Enuneku2015 | Lat (05^o^34'07.45''N),  Long (05^o^42'4.25''E) | Untreated and partly treated petrochemical wastes empty into Ubeji Creek and downstream to Warri river. | *Hemichromis fasciatus* |
| Ekpo2008 | - | Presence of Automobile workshops, car wash, battery charging, and other artisanal works. Domestic wastes empty into the river. | *Metacembelus Iconnbergii, Clarias lazera, Citarinus citharus, Tilapia zilli, Erpetoichthys calabaricus* |
| Edet2014 | Lat (5^o^ 39’ N),  Long (8^o^ 0’ E). | Site lies along a busy road. No heavy industry. Fishing, farming and petty trading as economic activities. | *Austropotamobius pallipes, Penaeus notialis,Tympanotonus fuscatus* |
| Daka2008 | - | Receives effluents from nearby industrial layout and abattoir. | *Liza falcipinnis, Sardinella madenensis, Tilapia mariae, Pomadasys jubelini, Gobius niger, Cynoglossus sp, Chrysichtyses nigodigitatus* |
| Ajima2015 | Lat (5°34′ - 5°38′ N),  Long (7°11′ - 7°12′ E) | Untreated effluents are released into Mbaa river from nearby aluminum industry. Other domestic wastes empty into the river. | *Pelmatochromis guentheri, Pelmatochromis pulcher* |
| Abarikwu2017 | - | Active oil exploitation and crude oil production around Ogale stream, effluents from petrochemical industries. Elele Elimini had limited industrial presence and low anthropogenic activity. | *Clarias gariepinus* |
| Alinnor 2005 | Lat (4^o^49 - 5^o^08N), Long (7.20E – 7.33E) | Effluents from nearby industries are discharged into the river. | *Lates niloticus, Hetretis niloticus, Oriochronis niloticus, Scombrus scombrus, Sadillina* |
| Akankali2018 | Long (6.92''E-6.94''E),  Lat (4⁰79'' - 4⁰80''N) | Receives domestic and industrial wastes. Presence of a maritime company. | *Mugil cephalus* |
| Edem2009 | - | Receives industrial and municipal waste effluents. | *Oreochromis niloticus* |
| Ediagbonya2019 | Lat (4^o^45’13.43” N),  Long (6^o^38’45.94”E) | Palm oil mill releases effluent into water body. Transportation, fishing, and sand mining. | *Tilapia zilli, Heterobranchus bidorsalis, Cyprinus carpio* |
| Nduka2010 | - | Active presence of a petrochemical industry, refinery and urban center. | *Clarias gariepinus, Oreochromis niloticus, Tilapia zilli, Serathrodon niloticus, Ethmaliosa timbriata* |
| Obasohan2007b | Lat (6° 20’ N), Long (5° 31’ E) | Presence of farmlands, car wash, brewery industry, rubber processing industry, and petroleum depot around sampling sites. | *Clarias gariepinus* |
| Obasohan2007a | Lat (6^o^50’N), Long (5^o^80’E) | Agricultural activities around sampling sites, river receives municipal wastes. | *Parachanna obscura* |

Table S2: Outcome from the subgroup meta-analysis of As

| As | | | | | |
| --- | --- | --- | --- | --- | --- |
| **Subgroup categories** | **Subgroups** | **Number of Study** | **PME (95% CI)** | **Heterogeneity (i.e. I^2^)** | **Chi 2 test for subgroup difference. (P values)** |
| **Seafood Types** | Fish | 7 | 0.2439 [-0.0835; 0.5714] | 98 % | p = 0.0820 |
|  | Shellfish | 4 | 1.4847 [ 0.1254; 2.8441] | 100 % |  |
| **Anthropogenic activities** | Non oil related industries | 3 | 0.0665 [-0.0057; 0.1387] | 93.5% | 0.0311 |
|  | Oil related industries | 7 | 1.0823 [ 0.1615; 2.0030] | 99.4% |  |
| **Sampling location (State)** | Rivers | 3 | 1.0263 [-0.5419; 2.5946] | 99.6% | < 0.0001 |
|  | Abia | 1 | 0.0088 [ 0.0078; 0.0098] | - |  |
|  | Delta | 3 | 0.9731 [-0.4358; 2.3819] | 99.1% |  |
|  | Ondo | 1 | 0.0950 [ 0.0813; 0.1087] | - |  |
|  | Imo | 1 | 1.6070 [ 1.1305; 2.0835] | - |  |
|  | Edo | 1 | 0.1250 [ 0.0833; 0.1667] | - |  |
| Study Year | 2005 and below | 1 | 0.0088 [ 0.0078; 0.0098] | - | < 0.0001 |
|  | 2006 - 2010 | 2 | 1.4142 [-0.4305; 3.2588] | 99.6% |  |
|  | 2011 - 2015 | 2 | 0.1185 [ 0.0952; 0.1417] | 0.0% |  |
|  | 2016 - 2022 | 5 | 0.9469 [-0.0910; 1.9848] | 99.2% |  |

Table S3: Outcome from the subgroup meta-analysis of Cd

| Cd | | | | | |
| --- | --- | --- | --- | --- | --- |
| **Subgroup categories** | **Subgroups** | **Number of Study** | **PME (95% CI)** | **Heterogeneity (i.e. I^2^)** | **Chi 2 test for subgroup difference. (P values)** |
| **Seafood Types** | Fish | 40 | 0.8848 [0.1519; 1.6177] | 100% | 0.5401 |
|  | Shellfish | 16 | 1.2139 [0.4581; 1.9697] | 100% |  |
| **Anthropogenic activities** | Domestic activities | 7 | 0.7638 [-0.0860; 1.6137] | 100% | 0.0593 |
|  | Urban/Commercial | 2 | 0.1073 [-0.0472; 0.2618] | 97.4% |  |
|  | Non-oil related industries | 9 | 0.2074 [ 0.0324; 0.3824] | 100.0% |  |
|  | Oil related industries | 33 | 1.1650 [ 0.2721; 2.0580] | 99.2% |  |
| **Sampling location (State)** | Rivers | 20 | 1.3510 [-0.0614; 2.7634] | 99.3% | < 0.0001 |
|  | Akwa Ibom | 4 | 0.8373 [-0.3837; 2.0582] | 99.8% |  |
|  | Bayelsa | 2 | 0.0357 [ 0.0355; 0.0359] | 0.0% |  |
|  | Cross River | 3 | 0.4026 [-0.2730; 1.0783] | 98.1% |  |
|  | Delta | 8 | 0.4047 [ 0.1571; 0.6523] | 99.0% |  |
|  | Ondo | 4 | 2.4633 [ 0.5831; 4.3435] | 100.0% |  |
|  | Imo | 2 | 0.8258 [ 0.4149; 1.2366] | 0.0% |  |
|  | Edo | 8 | 0.1809 [-0.0007; 0.3625] | 99.1% |  |
| **Study Year** | 2005 and below | 12 | 0.8420 [ 0.1688; 1.5152] | 100.0% | 0.0670 |
|  | 2006 - 2010 | 13 | 0.2334 [ 0.0573; 0.4095] | 99.3% |  |
|  | 2011 - 2015 | 12 | 0.7736 [ 0.1383; 1.4090] | 99.0% |  |
|  | 2016 - 2022 | 9 | 2.4003 [-0.5053; 5.3059] | 99.5% |  |

Table S4: Outcome from the subgroup meta-analysis of Co

| Co | | | | | |
| --- | --- | --- | --- | --- | --- |
| **Subgroup categories** | **Subgroups** | **Number of Study** | **PME (95% CI)** | **Heterogeneity (i.e. I^2^)** | **Chi 2 test for subgroup difference. (P values)** |
| **Seafood Types** | Fish | 12 | 1.9545 [ 0.1080; 3.8010] | 100.0% | < 0.0001 |
|  | Shellfish | 1 | 10.3559 [10.3557; 10.3561] | - |  |
| **Anthropogenic activities** | Domestic activities | 3 | 10.3559 [10.3557; 10.3561] | 0.0% | 0 |
|  | Non-oil related industries | 2 | 0.1495 [ 0.0047; 0.2942] | 98.8% |  |
|  | Oil related industries | 8 | 0.7264 [-0.0425; 1.4952] | 100.0% |  |
| **Sampling location (State)** | Rivers | 4 | 1.5521 [ 0.3120; 2.7921] | 98.2% | 0 |
|  | Abia | 1 | 0.0472 [ 0.0360; 0.0584] | - |  |
|  | Akwa Ibom | 1 | 0.0004 [ 0.0002; 0.0006] | - |  |
|  | Bayelsa | 1 | 0.0250 [ 0.0248; 0.0252] | - |  |
|  | Cross River | 1 | 0.0004 [ 0.0002; 0.0006] | - |  |
|  | Delta | 2 | 0.1350 [-0.0301; 0.3000] | 99.1% |  |
|  | Ondo | 3 | 10.3559 [10.3557; 10.3561] | 0.0% |  |
| **Study Year** | 2005 and below | 6 | 3.5097 [-0.2425; 7.2620] | 100% | < 0.0001 |
|  | 2011 - 2015 | 2 | 2.4279 [ 1.1331; 3.7228] | 92.5% |  |
|  | 2016 - 2022 | 1 | 10.3000 [ 8.9660; 11.6340] | - |  |

Table S5: Outcome from the subgroup meta-analysis of Cr

| Cr | | | | | |
| --- | --- | --- | --- | --- | --- |
| **Subgroup categories** | **Subgroups** | **Number of Study** | **PME (95% CI)** | **Heterogeneity (i.e. I^2^)** | **Chi 2 test for subgroup difference. (P values)** |
| **Seafood Types** | Fish | 29 | 2.0245 [1.0456; 3.0034] | 99.8% | 0.7506 |
|  | Shellfish | 11 | 1.7788 [0.6216; 2.9359] | 99.9% |  |
| **Anthropogenic activities** | Domestic activities | 4 | 1.1279 [-0.7870; 3.0427] | 99.9% | 0.6818 |
|  | Non-oil related industries | 7 | 2.1362 [ 0.4029; 3.8695] | 98.1% |  |
|  | Oil related industries | 28 | 2.0324 [ 1.0535; 3.0114] | 99.8% |  |
| **Sampling location (State)** | Rivers | 15 | 3.6055 [ 1.9870; 5.2240] | 99.9% | < 0.0001 |
|  | Edo | 3 | 0.4451 [ 0.2385; 0.6517] | 73.8% |  |
|  | Akwa Ibom | 4 | 0.2497 [-0.0131; 0.5125] | 99.9% |  |
|  | Bayelsa | 1 | 0.0004 [ 0.0002; 0.0006] | - |  |
|  | Cross River | 3 | 0.1118 [-0.0674; 0.2910] | 99.5% |  |
|  | Delta | 9 | 1.0792 [ 0.4592; 1.6992] | 98.8% |  |
|  | Imo | 1 | 0.0004 [ 0.0002; 0.0006] | - |  |
|  | Ondo | 3 | 3.0557 [ 0.5945; 5.5169] | 99.8% |  |
| **Study Year** | 2005 and below | 8 | 0.6710 [-0.0046; 1.3466] | 98.1% | 0.0197 |
|  | 2006 - 2010 | 7 | 1.6717 [ 0.4655; 2.8780] | 99.1% |  |
|  | 2011 - 2015 | 9 | 3.2131 [ 1.0230; 5.4033] | 99.9% |  |
|  | 2016 - 2022 | 10 | 2.7884 [ 1.1755; 4.4014] | 99.5% |  |

Table S6: Outcome from the subgroup meta-analysis of Cu

| Cu | | | | | |
| --- | --- | --- | --- | --- | --- |
| **Subgroup categories** | **Subgroups** | **Number of Study** | **PME (95% CI)** | **Heterogeneity (i.e. I^2^)** | **Chi 2 test for subgroup difference. (P values)** |
| **Seafood Types** | Fish | 33 | 8.9719 [2.5364; 15.4073] | 99.8% | 0.1568 |
|  | Shellfish | 12 | 17.0606 [7.8991; 26.2222] | 100.0% |  |
| **Anthropogenic activities** | Domestic activities | 6 | 13.2127 [-0.8878; 27.3133] | 100.0% | 0.1951 |
|  | Urban/Commercial | 1 | 5.9100 [ 4.4751; 7.3449] | - |  |
|  | Non-oil related industries | 11 | 4.4261 [ 1.1830; 7.6691] | 99.1% |  |
|  | Oil related industries | 24 | 13.6761 [ 4.6860; 22.6663] | 99.8% |  |
| **Sampling location (State)** | Rivers | 13 | 21.5270 [6.0087; 37.0453] | 99.9% | < 0.0001 |
|  | Edo | 6 | 5.2002 [1.1858; 9.2147] | 99.4% |  |
|  | Akwa Ibom | 3 | 4.1705 [0.7658; 7.5751] | 99.2% |  |
|  | Bayelsa | 1 | 5.4993 [4.2136; 6.7850] | - |  |
|  | Cross River | 4 | 2.8066 [0.0228; 5.5904] | 99.4% |  |
|  | Delta | 10 | 3.9215 [0.7513; 7.0918] | 98.9% |  |
|  | Imo | 2 | 1.3878 [1.1016; 1.6740] | 0.0% |  |
|  | Ondo | 3 | 22.9703 [2.4730; 43.4677] | 100.0% |  |
| **Study Year** | 2005 and below | 11 | 10.4409 [ 2.2768; 18.6051] | 100.0% | 0.8178 |
|  | 2006 - 2010 | 8 | 7.8618 [ 3.0906; 12.6330] | 98.6% |  |
|  | 2011 - 2015 | 11 | 9.5490 [ 3.0387; 16.0593] | 99.7% |  |
|  | 2016 - 2022 | 8 | 18.9657 [-7.0009; 44.9323] | 99.0% |  |

Table S7: Outcome from the subgroup meta-analysis of Fe

| Fe | | | | | |
| --- | --- | --- | --- | --- | --- |
| **Subgroup categories** | **Subgroups** | **Number of Study** | **PME (95% CI)** | **Heterogeneity (i.e. I^2^)** | **Chi 2 test for subgroup difference. (P values)** |
| **Seafood Types** | Fish | 30 | 138.3354 [47.2283; 229.4426] | 99.6% | 0.9072 |
|  | Shellfish | 9 | 129.2419 [ 6.3793; 252.1044] | 100.0% |  |
| **Anthropogenic activities** | Domestic activities | 6 | 387.9962 [-7.7540; 783.7465] | 100.0% | 0.1938 |
|  | Urban/Commercial | 1 | 110.6633 [74.1905; 147.1361] | - |  |
|  | Non-oil related industries | 5 | 66.2474 [-9.4787; 141.9734] | 99.4% |  |
|  | Oil related industries | 23 | 75.9251 [47.8189; 104.0313] | 99.2% |  |
| **Sampling location (State)** | Rivers | 9 | 108.4802 [ 48.6602; 168.3003] | 97.7% | 0.0049 |
|  | Edo | 1 | 40.1300 [ 32.7927; 47.4673] | - |  |
|  | Akwa Ibom | 28 | 28.4107 [ 1.0105; 55.8109] | 98.1% |  |
|  | Bayelsa | 1 | 27.0325 [ 20.6320; 33.4330] | - |  |
|  | Cross River | 4 | 191.8170 [ -1.5599; 385.1939] | 99.8% |  |
|  | Delta | 11 | 43.8128 [ 24.1024; 63.5231] | 92.5% |  |
|  | Imo | 2 | 45.8296 [ -0.4380; 92.0971] | 93.9% |  |
|  | Ondo | 4 | 509.7592 [-11.5348; 1031.0532] | 99.7% |  |
| **Study Year** | 2005 and below | 8 | 141.1888 [33.0595; 249.3181] | 100.0% | 0.0957 |
|  | 2006 - 2010 | 7 | 82.4895 [ 5.5735; 159.4055] | 99.8% |  |
|  | 2011 - 2015 | 8 | 42.1370 [18.4401; 65.8339] | 99.5% |  |
|  | 2016 - 2022 | 8 | 292.9034 [-7.0616; 592.8684] | 99.6% |  |

Table S8: Outcome from the subgroup meta-analysis of Hg

| Hg | | | | | |
| --- | --- | --- | --- | --- | --- |
| **Subgroup categories** | **Subgroups** | **Number of Study** | **PME (95% CI)** | **Heterogeneity (i.e. I^2^)** | **Chi 2 test for subgroup difference. (P values)** |
| **Seafood Types** | Fish | 8 | 0.0092 [ 0.0022; 0.0163] | 100.0% | 0.0558 |
|  | Shellfish | 4 | 0.0014 [-0.0025; 0.0052] | 83.5% |  |
| **Anthropogenic activities** | Domestic activities | 1 | 0.0001 [-0.0001; 0.0003] | - | 0.0034 |
|  | Non-oil related industries | 1 | 0.0110 [ 0.0022; 0.0198] | - |  |
|  | Oil related industries | 8 | 0.0096 [ 0.0017; 0.0176] | 100.0% |  |
| **Sampling location (State)** | Rivers | 3 | 0.0109 [ 0.0017; 0.0202] | 100.0% | 0.0 |
|  | Akwa Ibom | 2 | 0.0010 [-0.0003; 0.0023] | 97.2% |  |
|  | Bayelsa | 1 | 0.0286 [ 0.0284; 0.0288] | - |  |
|  | Cross River | 1 | 0.0004 [ 0.0002; 0.0006] | - |  |
|  | Delta | 2 | 0.0004 [ 0.0002; 0.0006] | 91.1% |  |
|  | Abia | 1 | 0.0110 [ 0.0022; 0.0198] | - |  |
| **Study Year** | 2005 and below | 3 | 0.0050 [ 0.0001; 0.0100] | 92.2% | 0.0001 |
|  | 2006 - 2010 | 2 | 0.0160 [ 0.0077; 0.0242] | 90.4% |  |
|  | 2011 - 2015 | 1 | 0.0001 [-0.0001; 0.0003] | - |  |

Table S9: Outcome from the subgroup meta-analysis of Mn

| Mn | | | | | |
| --- | --- | --- | --- | --- | --- |
| **Subgroup categories** | **Subgroups** | **Number of Study** | **PME (95% CI)** | **Heterogeneity (i.e. I^2^)** | **Chi 2 test for subgroup difference. (P values)** |
| **Seafood Types** | Fish | 27 | 8.4023 [3.5070; 13.2977] | 98.5% | 0.0823 |
|  | Shellfish | 6 | 43.4022 [4.2263; 82.5782] | 99.1% |  |
| **Anthropogenic activities** | Domestic activities | 4 | 41.5793 [-2.7301; 85.8886] | 99.8% | 0.0185 |
|  | Non-oil related industries | 9 | 1.0671 [ 0.7066; 1.4275] | 99.1% |  |
|  | Oil related industries | 18 | 11.4827 [ 2.1398; 20.8257] | 98.5% |  |
| **Sampling location (State)** | Rivers | 6 | 25.6902 [ -1.9145; 53.2950] | 97.2% | 0.0777 |
|  | Edo | 5 | 0.6832 [ 0.3557; 1.0107] | 97.4% |  |
|  | Akwa Ibom | 3 | 4.2186 [ -0.6223; 9.0594] | 99.7% |  |
|  | Bayelsa | 2 | 2.0882 [ -0.1010; 4.2773] | 97.6% |  |
|  | Cross River | 3 | 22.8597 [-11.1800; 56.8994] | 98.7% |  |
|  | Delta | 9 | 5.5478 [ -0.3021; 11.3977] | 97.5% |  |
|  | Abia | 1 | 0.6807 [ 0.5219; 0.8395] | - |  |
|  | Ondo | 2 | 52.3900 [-24.6505; 129.4305] | 97.0% |  |
| **Study Year** | 2005 and below | 9 | 18.3856 [-2.5541; 39.3254] | 99.5% | 0.1575 |
|  | 2006 - 2010 | 8 | 2.8420 [ 1.5548; 4.1293] | 94.4% |  |
|  | 2011 - 2015 | 5 | 27.8538 [-6.0352; 61.7428] | 99.5% |  |
|  | 2016 - 2022 | 4 | 6.1604 [ 0.0188; 12.3019] | 99.2% |  |

Table S10: Outcome from the subgroup meta-analysis of Ni

| Ni | | | | | |
| --- | --- | --- | --- | --- | --- |
| **Subgroup categories** | **Subgroups** | **Number of Study** | **PME (95% CI)** | **Heterogeneity (i.e. I^2^)** | **Chi 2 test for subgroup difference. (P values)** |
| **Seafood Types** | Fish | 32 | 5.6620 [2.8327; 8.4914] | 99.3% | 0.4643 |
|  | Shellfish | 10 | 7.6825 [3.0689; 12.2962] | 100.0% |  |
| **Anthropogenic activities** | Domestic activities | 3 | 8.5097 [-0.2876; 17.3070] | 100.0% | 0.0033 |
|  | Non-oil related industries | 9 | 1.4634 [ 0.1476; 2.7793] | 98.2% |  |
|  | Oil related industries | 28 | 6.5011 [ 3.5966; 9.4056] | 99.5% |  |
| **Sampling location (State)** | Rivers | 14 | 5.3848 [ 2.9993; 7.7702] | 99.5% | < 0.0001 |
|  | Edo | 5 | 0.2859 [ 0.2416; 0.3302] | - |  |
|  | Abia | 1 | 0.5817 [ 0.3626; 0.8008] | - |  |
|  | Akwa Ibom | 4 | 6.2738 [-0.1843; 12.7319] | 99.8% |  |
|  | Bayelsa | 2 | 0.1573 [ 0.1048; 0.2099] | 88.7% |  |
|  | Cross River | 1 | 9.1418 [ 7.9989; 10.2847] | - |  |
|  | Delta | 8 | 9.3709 [ 0.4469; 18.2950] | 96.9% |  |
|  | Imo | 1 | 1.2775 [ 0.8579; 1.6971] | - |  |
|  | Ondo | 4 | 6.9356 [-0.1211; 13.9923] | 100.0% |  |
| **Study Year** | 2005 and below | 13 | 5.9017 [ 3.0559; 8.7475] | 100.0% | 0.8218 |
|  | 2006 - 2010 | 9 | 5.2748 [ 1.1944; 9.3552] | 97.5% |  |
|  | 2011 - 2015 | 10 | 6.2665 [-0.9381; 13.4711] | 99.1% |  |
|  | 2016 - 2022 | 7 | 4.0641 [ 1.2008; 6.9274] | 99.6% |  |

Table S11: Outcome from the subgroup meta-analysis of Pb

| Pb | | | | | |
| --- | --- | --- | --- | --- | --- |
| **Subgroup categories** | **Subgroups** | **Number of Study** | **PME (95% CI)** | **Heterogeneity (i.e. I^2^)** | **Chi 2 test for subgroup difference. (P values)** |
| **Seafood Types** | Fish | 42 | 4.8190 [2.4642; 7.1738] | 100.0% | 0.3442 |
|  | Shellfish | 16 | 6.8285 [3.3947; 10.2623] | 100.0% |  |
| **Anthropogenic activities** | Domestic activities | 6 | 6.2290 [0.3985; 12.0594] | 100.0% | 0.0026 |
|  | Urban/Commercial | 2 | 0.6402 [-0.2962; 1.5767] | 99.2% |  |
|  | Non-oil related industries | 12 | 1.7355 [ 0.4898; 2.9811] | 100.0% |  |
|  | Oil related industries | 33 | 5.2424 [ 2.7102; 7.7745] | 97.1% |  |
| **Sampling location (State)** | Rivers | 19 | 5.5870 [ 2.1007; 9.0734] | 100.0% | 0.0131 |
|  | Edo | 8 | 0.8636 [ 0.0535; 1.6736] | 97.9% |  |
|  | Akwa Ibom | 4 | 1.1187 [-0.7639; 3.0014] | 99.3% |  |
|  | Bayelsa | 2 | 2.1222 [-0.3570; 4.6015] | 99.5% |  |
|  | Cross River | 4 | 5.2398 [-1.2775; 11.7572] | 99.5% |  |
|  | Delta | 10 | 4.4918 [ 0.1417; 8.8419] | 97.1% |  |
|  | Imo | 2 | 2.7392 [ 0.9337; 4.5446] | 87.6% |  |
|  | Ondo | 4 | 9.7283 [ 2.4015; 17.0550] | 100.0% |  |
| **Study Year** | 2005 and below | 17 | 5.7944 [ 2.8887; 8.7000] | 99.6% | 0.0080 |
|  | 2006 - 2010 | 13 | 1.4308 [ 0.6996; 2.1621] | 100.0% |  |
|  | 2011 - 2015 | 13 | 4.4204 [ 0.5779; 8.2629] | 99.9% |  |
|  | 2016 - 2022 | 9 | 6.4375 [-0.3067; 13.1817] | 99.6% |  |

Table S12: Outcome from the subgroup meta-analysis of Zn

| Zn | | | | | | | |
| --- | --- | --- | --- | --- | --- | --- | --- |
| **Subgroup categories** | | **Subgroups** | | **Number of Study** | **PME (95% CI)** | **Heterogeneity (i.e. I^2^)** | **Chi 2 test for subgroup difference. (P values)** |
| **Seafood Types** | | Fish | | 39 | 28.0298 [19.3636; 36.6960] | 99.9% | 0.1274 |
|  |  | Shellfish | | 9 | 57.7017 [20.5533; 94.8500] | 100.0% |  |
| **Anthropogenic activities** | | Domestic activities | | 7 | 31.5503 [ 4.2419; 58.8586] | 100.0% | 0.0001 |
|  |  | Urban/Commercial | | 1 | 55.7967 [41.8994; 69.6940] | - |  |
|  |  | Non-oil related industries | | 10 | 13.5013 [ 1.6210; 25.3816] | 99.9% |  |
|  |  | Oil related industries | | 27 | 32.3457 [21.1737; 43.5176] | 99.8% |  |
| **Sampling location (State)** | | Rivers | | 13 | 40.6734 [23.7402; 57.6067] | 99.6% | < 0.0001 |
|  |  | Edo | | 7 | 16.6373 [ 0.2599; 33.0148] | 99.9% |  |
|  |  | Abia | | 1 | 6.0017 [ 5.2526; 6.7508] | - |  |
|  |  | Akwa Ibom | | 5 | 12.2586 [ 3.1322; 21.3849] | 100.0% |  |
|  |  | Bayelsa | | 2 | 13.8337 [-3.1597; 30.8271] | 95.8% |  |
|  |  | Cross River | | 4 | 34.8804 [-4.6012; 74.3620] | 99.7% |  |
|  |  | Delta | | 8 | 25.8079 [10.6001; 41.0157] | 98.2% |  |
|  |  | Imo | | 2 | 10.9801 [-2.4808; 24.4410] | 99.4% |  |
|  |  | Ondo | | 3 | 58.5005 [13.8192; 103.1817] | 100.0% |  |
| **Study Year** | 2005 and below | | 11 | | 29.8424 [10.3067; 49.3782] | 100.0% | 0.6338 |
|  | 2006 - 2010 | | 9 | | 24.3395 [ 2.6580; 46.0210] | 100.0% |  |
|  | 2011 - 2015 | | 12 | | 36.6892 [16.6307; 56.7477] | 99.9% |  |
|  | 2016 - 2022 | | 8 | | 22.6616 [12.9441; 32.3792] | 98.6% |  |
